# Gut Microbiota Composition in Prediabetes and Newly Diagnosed Type 2 Diabetes: A Systematic Review of Observational Studies

**DOI:** 10.3389/fcimb.2022.943427

**Published:** 2022-08-15

**Authors:** Geetha Letchumanan, Natasya Abdullah, Muhamad Marlini, Nizam Baharom, Blair Lawley, Mohd Rahman Omar, Fathima Begum Syed Mohideen, Faizul Helmi Addnan, Mohd Manzor Nur Fariha, Zarini Ismail, Siva Gowri Pathmanathan

**Affiliations:** ^1^ Department of Medical Sciences, Faculty of Medicine & Health Sciences, Universiti Sains Islam Malaysia (USIM), Negeri Sembilan, Malaysia; ^2^ Public Health Unit, Department of Primary Health Care, Faculty of Medicine & Health Sciences, Universiti Sains Islam Malaysia (USIM), Negeri Sembilan, Malaysia; ^3^ Department of Microbiology and Immunology, School of Biomedical Sciences, University of Otago, Dunedin, New Zealand; ^4^ Medical-based Department, Faculty of Medicine & Health Sciences, Universiti Sains Islam Malaysia (USIM), Negeri Sembilan, Malaysia; ^5^ Family Medicine Unit, Department of Primary Health Care, Faculty of Medicine & Health Sciences, Universiti Sains Islam Malaysia (USIM), Negeri Sembilan, Malaysia

**Keywords:** gut microbiota, type 2 diabetes, prediabetes, 16S rRNA sequencing, systematic review

## Abstract

Evidence of gut microbiota involvement in regulating glucose metabolism and type 2 diabetes mellitus (T2DM) progression is accumulating. The understanding of microbial dysbiosis and specific alterations of gut microbiota composition that occur during the early stages of glucose intolerance, unperturbed by anti-diabetic medications, is especially essential. Hence, this systematic review was conducted to summarise the existing evidence related to microbiota composition and diversity in individuals with prediabetes (preDM) and individuals newly diagnosed with T2DM (newDM) in comparison to individuals with normal glucose tolerance (nonDM). A systematic search of the PubMed, MEDLINE and CINAHL databases were conducted from inception to February 2021 supplemented with manual searches of the list of references. The primary keywords of “type 2 diabetes”, “prediabetes”, “newly-diagnosed” and “gut microbiota” were used. Observational studies that conducted analysis of the gut microbiota of respondents with preDM and newDM were included. The quality of the studies was assessed using the modified Newcastle-Ottawa scale by independent reviewers. A total of 18 studies (5,489 participants) were included. Low gut microbial diversity was generally observed in preDM and newDM when compared to nonDM. Differences in gut microbiota composition between the disease groups and nonDM were inconsistent across the included studies. Four out of the 18 studies found increased abundance of phylum *Firmicutes* along with decreased abundance of *Bacteroidetes* in newDM. At the genus/species levels, decreased abundance of *Faecalibacterium prausnitzii*, *Roseburia*, *Dialister*, *Flavonifractor, Alistipes, Haemophilus* and *Akkermansia muciniphila* and increased abundance of *Lactobacillus, Streptococcus*, *Escherichi*a, *Veillonella* and *Collinsella* were observed in the disease groups in at least two studies. *Lactobacillus* was also found to positively correlate with fasting plasma glucose (FPG), HbA1c and/or homeostatic assessment of insulin resistance (HOMA-IR) in four studies. This renders a need for further investigations on the species/strain-specific role of endogenously present *Lactobacillus* in glucose regulation mechanism and T2DM disease progression. Differences in dietary intake caused significant variation in specific bacterial abundances. More studies are needed to establish more consistent associations, between clinical biomarkers or dietary intake and specific gut bacterial composition in prediabetes and early T2DM.

## Introduction

It is expected that by 2030, 578 million people worldwide will have diabetes, with type 2 diabetes mellitus (T2DM) accounting for about 90% of this staggering figure (Saeedi et al., 2019). With an estimated world population of 8,548 million by 2030, this would predict approximately 6% of the world’s population having T2DM by 2030 (Worldometers, 2021). In addition, approximately 8% of the world adult population is projected to have prediabetes by 2030, thereby also being at risk of developing full-blown T2DM (International Diabetes Federation (IDF), 2019). T2DM is a non-communicable disease characterized by an elevated blood glucose level or hyperglycaemia, defined by a fasting plasma glucose (FPG) level of ≥ 7.0mmol/l or 2-hour postprandial glucose level of ≥ 11.1mmol/l following oral glucose tolerance test (OGTT) (Alberti and Zimmet, 1998). Intermediate hyperglycaemia or prediabetes state, is defined by an impaired fasting glucose (IFG) and/or impaired glucose tolerance (IGT), after OGTT (Alberti and Zimmet, 1998). It was estimated that up to 21% of individuals with prediabetes will develop T2DM within three years (Eades et al., 2014).

Individuals with T2DM have an increased risk of developing complications such as kidney failure, retinopathy, neuropathy, cardiovascular disease and limb amputations (Centers for Disease Control Prevention, 2014). Recent evidence suggests a strong association between alterations in the gut microbiota composition and several metabolic disorders, including diabetes (Han and Lin, 2014). Several potential microbial molecular mechanisms have been suggested to contribute towards the onset and progression of T2DM (Gurung et al, 2020). On the other hand, use of metformin was found to be associated with modification in gut microbiota composition that contributed towards the therapeutic effects as well as known intestinal adverse effects of this most commonly used antidiabetic drug (Forslund et al., 2015). This emphasizes the need to detach observations of gut microbial alterations occurring in disease alone, free from the effects of drugs. As such, these specific changes in gut microbiota composition of prediabetic individuals and/or newly diagnosed diabetic individuals who have not begun pharmacotherapy, may serve as a predictive tool for identifying individuals at high-risk for developing T2DM. This would also enable future studies focusing on the specific microbial species to distinguish their role in disease as either cause or effect or both. This systematic review therefore aims to evaluate and summarise the existing evidence related to microbiota composition and diversity in individuals with prediabetes (preDM) and individuals newly diagnosed with T2DM (newDM) in comparison to individuals with normal glucose tolerance (nonDM). Findings on the association between the gut microbiota composition and clinical or dietary factors are also summarised.

## Methods

A systematic review of observational studies was performed according to a protocol published in the International Prospective Register of Systematic Reviews (PROSPERO) (CRD42020160458, 10/7/2020) (Pathmanathan et al., 2020) and reported according to the Preferred Reporting Items for Systematic Reviews and Meta-Analyses (PRISMA) guidelines (Moher et al., 2009).

### Search Strategy and Eligibility Criteria

A systematic search of published literature from inception to February 2021 was conducted using electronic databases including PubMed Central by the National Center for Biotechnology Information (PMC-NCBI), MEDLINE Complete and CINAHL (EBSCO Host). Briefly, main keywords included in the search were “type 2 diabetes”, “prediabetes”, “newly-diagnosed” and “gut microbiota”. The complete search strategy is provided in **Table 1**. Additional eligible studies were identified by hand-searching the reference lists of included studies. The search was limited to only studies published in English.

**Table 1:** Search terms and search strategy.

Search terms and search strategy		
type II diabetes/pre-diabetic	1	(((“type 2 diabet*”) OR “type II diabet*”)) OR type 2 diabetes[MeSH Terms]
	2	(((((prediabetes) OR prediabetics) OR prediabetic) OR pre-diabetes) OR pre-diabetics) OR pre-diabetic
	3	((“treatment naive”) OR “newly diagnosed”) OR “new diagnosis”
	4	(“impaired glucose tolerance”) OR “impaired fasting glucose”
	5	2 OR 3 OR 4 (((((((“treatment naive”) OR “newly diagnosed”) OR “new diagnosis”)) OR ((((((prediabetes) OR prediabetics) OR prediabetic) OR pre-diabetes) OR pre-diabetics) OR pre-diabetic)))) OR ((“impaired glucose tolerance”) OR “impaired fasting glucose”)
	6	1 AND 5 ((((((“type 2 diabet*”) OR “type II diabet*”)) OR type 2 diabetes[MeSH Terms]))) AND (((((“treatment naive”) OR “newly diagnosed”) OR “new diagnosis”)) OR ((((((prediabetes) OR prediabetics) OR prediabetic) OR pre-diabetes) OR pre-diabetics) OR pre-diabetic))
gut microbiota composition	7	(((microbiome) OR microbiota) OR microflora) OR “gut bacteria”
	8	6 AND 7 Search (((((((((“treatment naive”) OR “newly diagnosed”) OR “new diagnosis”)) OR ((((((prediabetes) OR prediabetics) OR prediabetic) OR pre-diabetes) OR pre-diabetics) OR pre-diabetic)))) OR ((“impaired glucose tolerance”) OR “impaired fasting glucose”))) AND (((((microbiome) OR microbiota) OR microflora) OR “gut bacteria”))
	9	Remove duplicates from 8

Studies that met the following criteria were included: (1) observational studies including case-control, cohort and cross-sectional study design; (2) studies on adult participants with newDM or preDM (3) studies that included a nonDM control group and (4) studies in which faecal samples were collected for gut microbial analysis. We excluded studies in which the participants were receiving treatment or dietary intervention prior to the investigation, studies in which participants provided microbiota samples from other body sites, animal studies and studies in which participants had other types of diabetes besides T2DM. Review articles, unpublished data, and articles in other languages were also excluded.

### Selection of Studies and Data Extraction

All search results were exported to a reference manager software (Endnote X9.3.1). Two authors (GL and SGP) selected the articles based on their titles and abstracts. Any disagreements between the reviewers were resolved through discussion with the third reviewer (NA). The full texts of eligible studies were assessed, and studies deemed irrelevant were excluded. A standard form was used to extract the data of included studies (**Supplementary Excel 1**).

Data recorded were general study characteristics (author name, year of publication, journal name, study design, country, sample size, gender distribution, mean age) and characteristics of methodology used by selected studies (microbiota quantification methods and diversity indices used) and the outcome measured. The primary outcomes were gut microbial abundance and differences between study groups at the phylum, class, order, family and genus taxonomic ranks. Species were grouped according to their respective genus. The secondary outcomes included clinical biomarkers, dietary intake and other parameters measured along with their correlation with the gut microbial composition.

### Quality Assessment

The study quality was assessed using the modified Newcastle Ottawa scale (Wells et al., 2014; Bjerre et al., 2017). The scale involves a maximum rating of nine stars divided into three categories: (1) samples selection, (2) comparability (comparison of the baseline parameters) and (3) exposure (defined measure of exposure and response rate between cases and controls). The studies were categorised based on their quality which were very good (score of 9); good (score of 7 to 8); fair (score of 5 to 6); and poor (score less than 5). Two researchers independently assessed the studies (GL and NB) and any discrepancies were resolved by another researcher (NA).

## Results

### Study Characteristics

**Figure 1:**
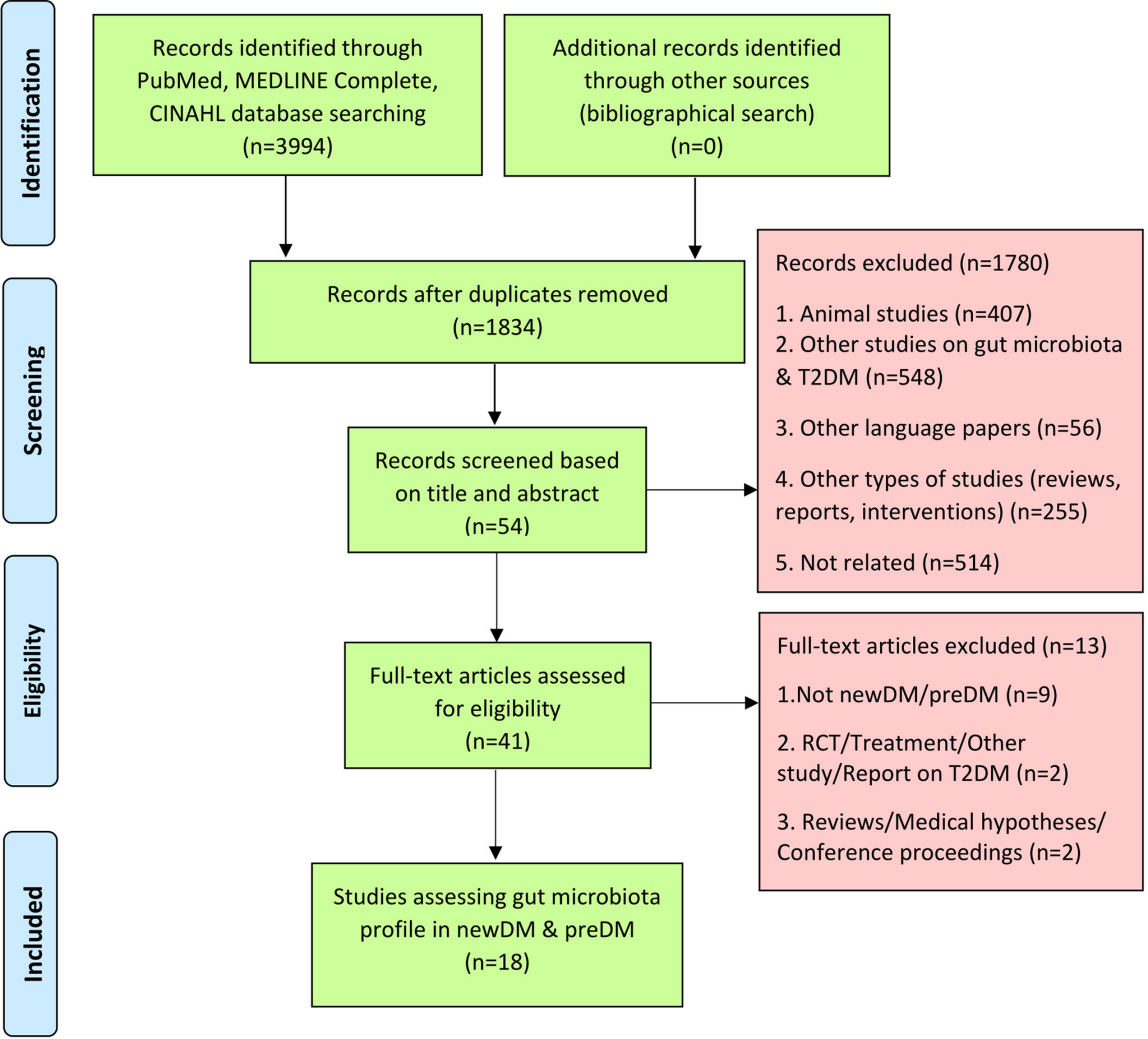
Flow chart of process undertaken to identify eligible studies, according to the PRISMA guidelines.

Of 3,994 articles identified, 18 studies were included in this systematic review (See **Figure 1** for PRISMA flow diagram). The characteristics of the study population are summarised in **Table 2**. The 18 observational studies had been conducted between 2013 and 2021. They included twelve case-control (Zhang et al., 2013; Lambeth et al., 2015; Bhute et al., 2017; Allin et al., 2018; Chen et al., 2019; Nuli et al., 2019; Zhao et al., 2019; Zhong et al., 2019; Gaike et al., 2020; Ghaemi et al., 2020; Li et al., 2020; Wang et al., 2021), four cross-sectional studies (Diener et al., 2021; Egshatyan et al., 2016; Chávez-Carbajal et al., 2020; Wu et al., 2020) and two cohort studies (Karlsson et al., 2013; Ericson et al., 2020). Six studies were conducted in Asia [India (Bhute et al., 2017; Gaike et al., 2020), Iran (Ghaemi et al., 2020), Taiwan (Chen et al., 2019) and China (Zhang et al., 2013; Nuli et al., 2019; Zhao et al., 2019; Zhong et al., 2019; Li et al., 2020; Wang et al., 2021)], five in Europe [Denmark (Allin et al., 2018), Russia (Egshatyan et al., 2016) and Sweden (Karlsson et al., 2013; Wu et al., 2020; Ericson et al., 2020)] and three in North America [USA (Lambeth et al., 2015) and Mexico (Diener et al., 2021; Chávez-Carbajal et al., 2020)]. These studies had compared the gut microbial profiles of either preDM alone (Allin et al., 2018; Ericson et al., 2020), newDM alone (Chen et al., 2019; Li et al., 2020), preDM and newDM (Zhang et al., 2013; Egshatyan et al., 2016; Nuli et al., 2019; Zhong et al., 2019; Wu et al., 2020), preDM and knownDM (Karlsson et al., 2013; Lambeth et al., 2015; Ghaemi et al., 2020; Chávez-Carbajal et al., 2020; Wang et al., 2021), newDM and knownDM (Bhute et al., 2017; Zhao et al., 2019) or all three preDM, newDM and knownDM (Diener et al., 2021; Gaike et al., 2020) in comparison to gut microbial profile of nonDM. The findings on knownDM were not analysed in this review.

**Table 2:** Summary of study characteristics.

No.	Study Reference	Type of Study	Country	Sample Size, n	Age group (average)	Ethnicity	No. of Subjects, n (female/male)					
							preDM				newDM,	Controls
							IGT	IFG		CGI		
1	(Allin et al., 2018)	Case-control	Denmark	268	55-68	Danish		134 (53/81)				134 (53/81)
2	(Bhute et al., 2017)	Case-control	India	49	40-60	Indian					14	19
3	(Chávez-Carbajal et al., 2020)	Cross-sectional	Mexico	217	40-63	Mexicans		54 (36/18)				76 (50/26)
4	(Chen et al., 2019)	Case-control	Taiwan	100	20-80	N/A					50 (14/36)	50 (22/28)
5	(Diener et al., 2021)	Cross-sectional	Mexico	430	24-66	Mexicans	42 (29/13)	52 (29/23)		57 (39/18)	48 (31/17)	214 (165/49)
6	(Egshatyan et al., 2016)	Cross-sectioal	Russia	97	25-75	Caucasian	25 (18/7)				23 (13/10)	49 (38/11)
7	(Ericson et al., 2020)	Cohort	Sweden	1726	>18	N/A		260 (137/123)				1466 (800/666)
8	(Gaike et al., 2020)	Case-control	India	102	30-60	Indian		17 (11/6)			11 (2/9)	35 (18/17)
9	(Ghaemi et al., 2020)	Case-control	Iran	90	40-60	Iranian	30					30
10	(Karlsson et al., 2013)	Cohort	Sweden	145 (all women)	70	European	49					43
11	(Lambeth et al., 2015)	Case-control	USA	49	55-62	Caucasian white, Hispanics, Native Americans		20 (14/6)				15 (10/5)
12	(Li et al., 2020)	Case-control	China	60	40 -50	N/A					30 (26/4)	30 (26/4)
13	(Nuli et al., 2019)	Case-control	China	60	30-70	Chinese (Uyghur)	20 (8/12)				20 (9/11)	20 (8/12)
14	(Wang et al., 2021)	Case-control	China	126	40-70	Chinese	33 (22/11)					63 (40/23)
15	(Wu et al., 2020)	Cross-sectional	Sweden	1495	50-64	Swedish	DC (178) [98/80] VC (132) [74/58]	DC (189) [98/91]		DC (75)[28/47] VC (88)[39/49]	DC (46) [17/29] VC (58) [27/32]	DC:523 [lrNGT: 226 (127/99); hrNGT: 297: (200/97)] VC:206 (100/106)
16	(Zhang et al., 2013)	Case-control	China	121	50-55	N/A	64				13	44
17	(Zhao et al., 2019)	Case-control	China	100	40-60	Chinese					16 (9/7)	35 (17/18)
18	(Zhong et al., 2019)	Case-control	China	254	49-75	Chinese	80 (39/41)				77 (44/33)	97 (65/32)

DC, discovery cohort; VC, validation cohort; lrNGT, low-risk NGT; hrNGT, high-risk NGT.

The 18 studies consisted of 5,489 participants. In 15 of the 18 studies, 43% of the participants were male (n=2, 252) and 57% were female (n= 2,978). Three studies (Zhang et al., 2013; Bhute et al., 2017; Ghaemi et al., 2020) did not specify the participants’ gender. The mean age of participants was 50 ± 7.82 years. There was a total of 3,149 participants in the control or nonDM group. The remaining 2,340 participants with varying glucose levels included 1,599 preDM, 406 newDM and 335 knownDM. PreDM was diagnosed using either IFG (Lambeth et al., 2015; Allin et al., 2018; Gaike et al., 2020; Chávez-Carbajal et al., 2020; Ericson et al., 2020), IGT (Karlsson et al., 2013), combined glucose intolerance (CGI) (Wu et al., 2020), IFG and/or IGT (Diener et al., 2021; Zhang et al., 2013; Egshatyan et al., 2016; Zhong et al., 2019; Ghaemi et al., 2020; Wu et al., 2020; Wang et al., 2021). NewDM was diagnosed using OGTT (Diener et al., 2021; Zhang et al., 2013; Egshatyan et al., 2016; Bhute et al., 2017; Nuli et al., 2019; Zhao et al., 2019; Zhong et al., 2019; Li et al., 2020; Wu et al., 2020), HbA1c (Egshatyan et al., 2016; Zhao et al., 2019; Gaike et al., 2020) and fasting plasma glucose (FPG) (Chen et al., 2019). In 10 out of 11 studies that had participants with newDM, no information was provided regarding period between newDM diagnosis to sample collection while one study reported to only have included participants with newDM who had disease duration of <12 months and a HbA1c range of 6.5 – 9.0% (Egshatyan et al., 2016).

### Quality Assessment for Risk of Bias

**Table S1** summarises the quality assessment of the studies included. The mean score for the studies included was 8 (range of 6-9) out of a possible total of 9. All studies received either very good or good scores. One study received three out of five maximum scores in the section on study selection as it did not describe sampling strategy or justify the sample size (Chávez-Carbajal et al., 2020), which are criteria to be fulfilled for cross sectional studies, based on the decision rule in the modified Newcastle Ottawa scale used in this review. In the comparability section, five studies (Zhang et al., 2013; Bhute et al., 2017; Chen et al., 2019; Gaike et al., 2020; Ghaemi et al., 2020) stated that cases and controls were matched based on diabetes status alone and did not take into account other confounding factors such as gender or age. Meanwhile, in the exposure section, 11 studies (Zhang et al., 2013; Lambeth et al., 2015; Bhute et al., 2017; Allin et al., 2018; Chen et al., 2019; Nuli et al., 2019; Zhao et al., 2019; Zhong et al., 2019; Gaike et al., 2020; Ghaemi et al., 2020; Li et al., 2020) did not mention the number of study participants who dropped out, if any.

### Microbiome Analysis Methods

**Table 3** summarises the methodologies adapted by the studies to analyse the microbiome data. These studies used varying DNA kits for DNA extraction and different microbiome sequencing platforms including Illumina Miseq (Diener et al., 2021; Lambeth et al., 2015; Egshatyan et al., 2016; Allin et al., 2018; Nuli et al., 2019) or Hi-Seq (Karlsson et al., 2013; Gaike et al., 2020; Wu et al., 2020; Ericson et al., 2020; Wang et al., 2021) sequencing platforms, the 454 GS FLX Titanium pyro-sequencer (Zhang et al., 2013), PGM sequencing (Ion Touch 2) (Bhute et al., 2017; Chávez-Carbajal et al., 2020), Ion S5 sequencer with Ion Torrent Technology (Zhao et al., 2019), shotgun metagenomics sequencing using the BGISEQ-500 platform (Zhong et al., 2019) as well as quantitative polymerase chain reaction (qPCR) (Chen et al., 2019; Ghaemi et al., 2020). When the 16S rDNA was targeted for sequencing, the hypervariable regions targeted included V4 (Diener et al., 2021; Lambeth et al., 2015; Allin et al., 2018; Gaike et al., 2020), V3-V4 (Egshatyan et al., 2016; Nuli et al., 2019; Zhao et al., 2019), V3 (Bhute et al., 2017; Chávez-Carbajal et al., 2020), V1-V3 (Ericson et al., 2020) or V3-V5 (Zhang et al., 2013) regions, while five studies did not specify the target regions (Karlsson et al., 2013; Zhong et al., 2019; Li et al., 2020; Wu et al., 2020; Wang et al., 2021). The two studies using qPCR to assess the microbiota had used ten and six pairs of specific bacterial 16S rRNA primers, respectively, to target *Atopobium cluster, Bacteroides fragilis, Bifidobacterium, Clostridium coccoides, Clostridium leptum, Clostridium perfringens, Enterobacteriaceae, Enterococcus, Lactobacillus* and *Prevotella* (Chen et al., 2019) and *Akkermansia, Bacteroides, Bifidobacterium, E.coli, Faecalibacterium and Lactobacillus* (Ghaemi et al., 2020).

**Table 3:** Characteristics of the methodology used by the 18 selected articles for gut microbiota composition and diversity assessments.

No.	Study Reference	DNA extraction kit/method		Gut microbiota amplification region and sequencing platform used	Taxonomical classification	Gut microbiota diversity assessment measures	
						α-diversity index	β-diversity index
1	(Allin et al., 2018)	NucleoSpin Soil Mini Kit, Macharey-Nagel		16S rRNA V4 region - Illumina Miseq	OTU	Observed OTUs and Phylogenetic Diversity	Unweighted UniFrac and PCoA
2	(Bhute et al., 2017)	QIAmp DNA Stool Mini Kit, Qiagen		Eubacterial 16S rRNA, Archaeal 16S, Eukaryotic 18S and fungal ITS genes- Ion Torrent PGM	OTU	Observed OTUs and Chao1	Weighted, Unweighted UniFrac and PCoA
3	(Chávez-Carbajal et al., 2020)	MoBio PowerSoil DNA Isolation Kit, Mo Bio Laboratories		16S rRNA V4 region – Ion Torrent PGM	OTU	Observed OTUs, Chao1, Shannon and Simpson	Unweighted UniFrac and PCoA
4	(Chen et al., 2019)	QIAamp Fast DNA Stool Mini Kit, Qiagen		specific bacterial 16S rRNA primers -quantitative polymerase chain reaction (qPCR)	Microbiota (log10 cell/g)	Not stated	Not stated
5	(Diener et al., 2021)	MoBio PowerSoil DNA Isolation Kit, Mo Bio Laboratories	16S rRNA V4 region - Illumina Miseq		ASV	Shannon	Not stated
6	(Egshatyan et al., 2016)	Chemical-based method	16S rRNA V3-V4 region – Illumina Miseq		OTU	Not stated	UniFrac and Multidimensional Scaling (MDS) plot
7	(Ericson et al., 2020)	QIAmp DNA Stool Mini Kit, Qiagen	16S rRNA V1-V3 region – Illumina HiSeq		OTU	Shannon	Not stated
8	(Gaike et al., 2020)	QIAmp DNA Stool Mini Kit, Qiagen	16S rRNA V4 region - Illumina HiSeq		OTU	Observed OTUs and Simpson	Weighted and Unweighted UniFrac
9	(Ghaemi et al., 2020)	QIAmp DNA Stool Mini Kit, Qiagen	specific bacterial 16S rRNA primers -qPCR		Log10 CFU/g stool	Not stated	Not stated
10	(Karlsson et al., 2013)	QIAmp DNA Stool Mini Kit, Qiagen	Illumina HiSeq 2000		Metagenomic Clusters (MGC)	Not stated	Not stated
11	(Lambeth et al., 2015)	QIAmp DNA Stool Mini Kit, Qiagen	16S rRNA V4 region - Illumina MiSeq		OTU	Shannon	Bray-Curtis, Unweighted and Weighted UniFrac
12	(Li et al., 2020	MoBio PowerSoil DNA Isolation Kit, Mo Bio Laboratories	16S rRNA full length - Illumina Nova		OTU	ACE, Chao1, Shannon and Simpson	Not stated
13	(Nuli et al., 2019)	QIAmp DNA Stool Mini Kit, Qiagen	16S rRNA V3-V4 region - Illumina Miseq		OTU	ACE, Chao1, Shannon, Simpson and Sobs	Not stated
14	(Wang et al., 2021)	Sodium dodecyl sulfate (SDS) method	Illumina HiSeq		Metagenome	Shannon	Not stated
15	(Wu et al., 2020)	Repeated bead beating method	Illumina Hiseq		MGC	Not stated	Bray-Curtis and PCoA
16	(Zhang et al., 2013)	Commercial kit, iNtRON Biotechnology	16S rRNA V3-V5 region - 454 GS FLX Titanium pyro-sequencer		OTU	Chao1 and Shannon	Principal component analysis (PCA)
17	(Zhao et al., 2019)	FastDNA Spin Kit, MP Biomedicals	16S rRNA V3-V4 region - Ion S5 sequencer		OTU	Chao1, Shannon and Simpson	Unweighted UniFrac and PCoA
18	(Zhong et al., 2019)	Chemical-based method	Shotgun metagenomic sequencing - BGISEQ-500		Metagenomic Linkage Groups (MLG)	Shannon	Bray-Curtis

Following sequencing, the microorganisms were classified and reported as operational taxonomic units (OTUs) (Zhang et al., 2013; Lambeth et al., 2015; Egshatyan et al., 2016; Bhute et al., 2017; Allin et al., 2018; Nuli et al., 2019; Zhao et al., 2019; Gaike et al., 2020; Li et al., 2020; Chávez-Carbajal et al., 2020; Ericson et al., 2020), metagenomic clusters (MGCs) (Karlsson et al., 2013; Wu et al., 2020), microbiota (log10 cell/g) (Chen et al., 2019; Ghaemi et al., 2020), metagenome (Wang et al., 2021), metagenomic linkage group (MLGs) (Zhong et al., 2019) or amplicon sequence variants (ASVs) (Diener et al., 2021).

### Diversity in Gut Microbiota

The α-diversity or the average microbial diversity within a single sample was reported by 13 of the 18 studies and the key measures used were Shannon, Simpson, observed species richness, abundance-based coverage estimator (ACE), Chao1 and phylogenetic diversity indexes (**Table 3**).

Six of the studies found a statistically significant lower α-diversity in the disease groups namely preDM (Allin et al., 2018; Chávez-Carbajal et al., 2020), newDM (Zhang et al., 2013; Bhute et al., 2017; Gaike et al., 2020) and in both preDM and newDM (Nuli et al., 2019) in comparison to nonDM. Two other studies reported a non-significant reduction in α-diversity in preDM (Lambeth et al., 2015) and newDM (Zhao et al., 2019). On the other hand, another two studies, (Zhong et al., 2019) and (Wang et al., 2021) found no significant difference in α-diversity among preDM and newDM in comparison to nonDM. Diener et al. (2021) found that genera *Ruminococcaceae* was the most positively correlated with α-diversity while *Fusobacterium*, *Flavonifractor*, and *Parasutterella* were the most negatively correlated with α-diversity (Diener et al., 2021). Allin et al. (2018) went on to demonstrate that α-diversity in the total group of subjects was negatively correlated with T2DM biomarkers particularly plasma triacylglycerol and high sensitivity C-reactive protein (hsCRP), as well as body mass index (BMI), hip circumference (HC), fasting blood glucose (FBG), fasting plasma insulin (FINS), plasma C-peptide and HOMA-IR (Allin et al., 2018). Ericson et al. (2020) examined the correlation between the food patterns and α-diversity in preDM and nonDM, but discovered no statistically significant association (Ericson et al., 2020).

The β-diversity or the measure of how gut microbial composition vary between study groups was reported by 11 studies using principal coordinate analysis (PCoA), principal component analysis (PCA) of either weighted or unweighted UniFrac or Bray Curtis dissimilarities distance matrices (**Table 3**).

Six studies found a significant difference in β-diversity among preDM (Chávez-Carbajal et al., 2020), newDM (Bhute et al., 2017; Zhao et al., 2019; Li et al., 2020) and in both preDM and newDM (Nuli et al., 2019; Wu et al., 2020) in comparison to nonDM. Four studies found no difference in the β-diversity between disease groups and nonDM groups (Zhang et al., 2013; Allin et al., 2018; Zhong et al., 2019; Wang et al., 2021). Gaike et al. (2020) observed using Principal Coordinate Analysis (PCoA), that bacterial diversity of newDM was distinct from that of nonDM, whereas preDM formed an overlapping cluster with nonDM indicating similarity in bacterial diversity (Gaike et al., 2020).

### Gut Microbiota Composition

All eighteen studies provided information on microbial abundance by genus/species ranks. Eleven studies (Zhang et al., 2013; Karlsson et al., 2013; Lambeth et al., 2015; Egshatyan et al., 2016; Bhute et al., 2017; Nuli et al., 2019; Zhao et al., 2019; Zhong et al., 2019; Gaike et al., 2020; Li et al., 2020; Chávez-Carbajal et al., 2020) reported the taxonomic rank of microbial abundance by phylum level. Nine studies (Zhang et al., 2013; Karlsson et al., 2013; Lambeth et al., 2015; Bhute et al., 2017; Allin et al., 2018; Nuli et al., 2019; Zhao et al., 2019; Li et al., 2020; Chávez-Carbajal et al., 2020) reported by ranks of class, order and family.

Eight studies reported that *Bacteroidetes* and *Firmicutes* were the predominant phyla in all groups studied, i.e., preDM and/or newDM and nonDM (Zhang et al., 2013; Karlsson et al., 2013; Lambeth et al., 2015; Egshatyan et al., 2016; Nuli et al., 2019; Zhao et al., 2019; Zhong et al., 2019; Li et al., 2020). *Proteobacteria* was reported as the next predominant phyla in five out of these eight studies (Karlsson et al., 2013; Lambeth et al., 2015; Nuli et al., 2019; Zhao et al., 2019; Zhong et al., 2019). All eighteen studies reported significant differences in gut microbiota composition by microbial taxa in the disease groups i.e. preDM and/or newDM when compared to the nonDM control group (**Table 4** and **Table S2**). In four studies, a significant increase in the phylum *Firmicutes* along with a significant decrease in phylum *Bacteroidetes* were observed in the newDM group (Bhute et al., 2017; Nuli et al., 2019; Zhao et al., 2019; Gaike et al., 2020). Two of these four studies each found a significant increase (Zhao et al., 2019; Gaike et al., 2020) or significant decrease (Bhute et al., 2017; Nuli et al., 2019) in *Proteobacteria* respectively. Meanwhile, two other studies reported a significant decrease in phylum *Verrucomicrobia* in preDM group (Zhang et al., 2013; Egshatyan et al., 2016). Two studies reported increased *Firmicutes/Bacteroidetes* ratio (*F/B* ratio) among newDM (Zhao et al., 2019; Li et al., 2020) and one study reported increased *F/B* ratio among both preDM and newDM (Gaike et al., 2020).

**Table 4:** The changes (increase or decrease) noted in gut microbiota of preDM and newDM in comparison to nonDM by microbial taxa and number of reporting studies. All findings are significant (p <0.050).

Taxa level	Increased in ≥ 3 papers		Increased in 2 papers		Increased in 1 paper		Decreased in 1 paper		Decreased in 2 papers		Decreased in ≥ 3 papers	
	preDM	newDM	preDM	newDM	preDM	newDM	preDM	newDM	preDM	newDM	preDM	newDM
**Phylum**		*Firmicutes* (Bhute et al., 2017; Gaike et al., 2020; Nuli et al., 2019; Zhao et al., 2019)		*Proteobacteria* (Gaike et al., 2020; Zhao et al., 2019)	*Actinobacteria* (Nuli et al., 2019) *Bacteroidetes* (Lambeth et al., 2015) *Firmicutes* (Allin et al., 2018; Nuli et al., 2019) *Saccharibacteria* (Nuli et al., 2019)	*Actinobacteria* (Nuli et al., 2019) *Saccharibacteria* (Nuli et al., 2019)	*Bacteroidetes* (Nuli et al., 2019) *Firmicutes* (Lambeth et al., 2015) *Proteobacteria* (Nuli et al., 2019)	*Verrucomicrobia* (Gaike et al., 2020)	*Verrucomicrobia* (Egshatyan et al., 2016; Zhang et al., 2013)	*Proteobacteria* (Bhute et al., 2017; Nuli et al., 2019)		*Bacteroidetes* (Bhute et al., 2017; Gaike et al., 2020; Nuli et al., 2019; Zhao et al., 2019)
**Class**					*Betaproteobacteria* (Zhang et al., 2013) *Deferribacteres* (Nuli et al., 2019)	*Betaproteobacteria* (Zhang et al., 2013) *Clostridia* (Zhang et al., 2013) *Deferribacteres* (Nuli et al., 2019)	*Betaproteobacteria* (Chávez-Carbajal et al., 2020)	*Bacteroidia* (Li et al., 2020) *Betaproteobacteria* (Chávez-Carbajal et al., 2020)				
**Order**					*Clostridiales* (Karlsson et al., 2013)	*Clostridiales* (Zhang et al., 2013) *Selenomonadales* (Li et al., 2020) *Veillonellales* (Li et al., 2020)	*Burkholderiales* (Chávez-Carbajal et al., 2020)	*Acidaminococcales* (Li et al., 2020) *Burkholderiales* (Chávez-Carbajal et al., 2020)		*Bacteroidales* (Li et al., 2020; Nuli et al., 2019)	*Clostridiales* (Allin et al., 2018; Karlsson et al., 2013; Nuli et al., 2019)	
**Family**				*Lachnospiraceae* (Nuli et al., 2019; Zhang et al., 2013)	*Comamonadacea*e (Chávez-Carbajal et al., 2020) *Pasteurellaceae* (Nuli et al., 2019) *Pseudonocardiaceae* (Lambeth et al., 2015)	*Ruminococcaceae* (Nuli et al., 2019) *Selenomonadaceae* (Li et al., 2020)	*Alcaligenaceae* (Chávez-Carbajal et al., 2020) *Clostridiaceae* (Allin et al., 2018) *Christensenellaceae* (Allin et al., 2018) *Porphyromonadaceae* (Nuli et al., 2019) *Rikenellaceae* (Allin et al., 2018)	*Acidaminococcaceae* (Li et al., 2020) *Alcaligenaceae* (Chávez-Carbajal et al., 2020) *Lachnospiraceae* (Bhute et al., 2017) *Porphyromonadaceae* (Nuli et al., 2019) *Prevotellaceae* (Bhute et al., 2017) *Ruminococcacea* (Bhute et al., 2017)	*Coriobacteriaceae* (Karlsson et al., 2013; Nuli et al., 2019) *Lachnospiraceae* (Allin et al., 2018; Karlsson et al., 2013) *Ruminococcaceae* (Allin et al., 2018; Nuli et al., 2019)			
**Genus/ Species**	*Escherichia* (Diener et al., 2021) *Esherichia coli* (Ghaemi et al., 2020; Zhong et al., 2019) *Streptoco-ccus* (Allin et al., 2018) *Streptoco-ccus mutans* (Karlsson et al., 2013) *Streptoco-ccus salivarius* (Zhong et al., 2019) *Streptococ-cus thermophilus* (Allin et al., 2018)	*Lactobacillus* (Bhute et al., 2017; Chen et al., 2019; Gaike et al., 2020) *Lactobacillus ruminis* (Bhute et al., 2017)	*Bacteroides fragilis* (Ghaemi et al., 2020) *Bacteroides uniformis* (Allin et al., 2018) *Blautia* (Allin et al., 2018; Egshatyan et al., 2016) *Blautia wexlerae* (Allin et al., 2018) *Clostridium boltae* (Wu et al., 2020) *Clostridium clostridioforme* (Karlsson et al., 2013; Wu et al., 2020) *Prevotella* (Egshatyan et al., 2016; Zhang et al., 2013) *Veillonella* (Diener et al., 2021; Nuli et al., 2019)	*Blautia* (Egshatyan et al., 2016; Zhao et al., 2019) *Collinsella* (Zhang et al., 2013) *Collinsella intestinalis* (Zhong et al., 2019) *Coprococcus 1* (Zhao et al., 2019) *Coprococcus eutactus* (Zhong et al., 2019) *Eubacterium* (Zhang et al., 2013) *Eubacterium halii* (Zhao et al., 2019) *Prevotella* (Egshatyan et al., 2016; Zhang et al., 2013)	*Chloracidobacteria* (Lambeth et al., 2015) *Coprococcus comes* (Allin et al., 2018) *Dorea* (Allin et al., 2018) *Dorea longicatena* (Allin et al., 2018) *Eggerthella sp* (Zhong et al., 2019) *Faecalibacterium prausnitzii* (Allin et al., 2018) *Haemophilus* (Nuli et al., 2019) *Lachnospira* (Ericson et al., 2020) *Lactobacillus* (Karlsson et al., 2013) *Lactobacillus gasseri* (Karlsson et al., 2013) *Lactobacillus salivarius* (Karlsson et al., 2013) *Megamonas* (Nuli et al., 2019) *Megasphaera elsdenii* (Zhong et al., 2019) *Roseburia* (Ericson et al., 2020) *Ruminococcus* (Allin et al., 2018) *Ruminococcus gnavus* (Allin et al., 2018) *Ruminococcus torques* (Allin et al., 2018) *Serratia* (Egshatyan et al., 2016) *Sutterella* (Allin et al., 2018)	*Abiotrophia* (Zhang et al., 2013) *Bacteroides caccae* (Zhong et al., 2019) *Bacteroides finegoldii* (Zhong et al., 2019) *Cetobacterium* (Nuli et al., 2019) *Clostridium boltae* (Wu et al., 2020) *Clostridium clostridioforme* (Wu et al., 2020) *Dorea* (Zhang et al., 2013) *Escherichia* (Diener et al., 2021) *Faecalibacterium prausnitzii* (Zhong et al., 2019) *Lachnospira* (Nuli et al., 2019) *Megamonas* (Li et al., 2020) *Megamonas funiformis* (Li et al., 2020) *Megapshaera elsdenii* (Zhong et al., 2019) *Mucispirillum* (Nuli et al., 2019) *Peptostreptococcus* (Zhang et al., 2013) *Proteiniphilum* (Nuli et al., 2019) *Ruminococus* (Zhang et al., 2013) *Serratia* (Egshatyan et al., 2016) *Sporobacter* (Zhang et al., 2013) *Streptococcus* (Bhute et al., 2017) *Subdoligranulum* (Zhang et al., 2013) *Sutterella* (Chávez-Carbajal et al., 2020) *Tyzzerella* (Nuli et al., 2019) *Veillonella* (Diener et al., 2021)	*Anaerostipes* (Diener et al., 2021) *Anaerotruncus* (Ericson et al., 2020) *Barnesiella* (Nuli et al., 2019) *Bilophila* (Allin et al., 2018) *Clostridiales bacterium* (Wu et al., 2020) *Dialister invisus* (Zhong et al., 2019) *Dielma* (Nuli et al., 2019) *Dorea longicatena* (Karlsson et al., 2013) *Desulfurispirillum indicum* (Karlsson et al., 2013) *Haemophilus parainfluenza* (Zhong et al., 2019) *Intestinimonas butyriciproducens* (Wu et al., 2020) *Klebsiella oxytoca* (Wang et al., 2021) *Lachnospira* (Allin et al., 2018) *Oscillibacter spp.* (Wu et al., 2020) *Pseudoflavonifractor spp*. (Wu et al., 2020) *Pyramidobacter piscolens* (Karlsson et al., 2013) *Ruminococcus* (Nuli et al., 2019) *Ruminiclostridium* (Nuli et al., 2019) *Sutterella* (Nuli et al., 2019) *Streptococcus* (Zhang et al., 2013)	*Anaerostipes* (Diener et al., 2021) *Alistipes sp.* (Wu et al., 2020) *Barnesiella* (Nuli et al., 2019) *Coprococcus sp*. (Zhong et al., 2019) *Dielma* (Nuli et al., 2019) *Megamonas* (Zhang et al., 2013) *Oscillibacter sp.* (Wu et al., 2020) *Phascolarctobacterium faecium* (Li et al., 2020) *Pseudoflavonifractor sp*. (Wu et al., 2020) *Ruminococcus* (Gaike et al., 2020) *Ruminoclostridium* (Nuli et al., 2019) *Streptococcus* (Zhang et al., 2013)	*Akkermansia muciniphila* (Allin et al., 2018; Zhang et al., 2013) *Alistipes* (Karlsson et al., 2013) *Alistipes obesi* (Karlsson et al., 2013) *Alistipes sp* (Wu et al., 2020) *Bacteroides* (Allin et al., 2018) *Bacteroides intestinalis* (Karlsson et al., 2013) *Blautia* (Allin et al., 2018; Diener et al., 2021) *Coprococcus* (Allin et al., 2018) *Coprococcus eutactus* (Wu et al., 2020) *Coprococcus sp.* (Zhong et al., 2019) *Eubacterium* (Ericson et al., 2020) *Eubacterium eligens* (Karlsson et al., 2013) *Flavonifractor* (Nuli et al., 2019) *Flavonifractor plautii* (Wu et al., 2020) *Roseburia* (Karlsson et al., 2013) *Roseburia hominis* (Zhong et al., 2019)	*Akkermansia* (Gaike et al., 2020) *Akkermansia muciniphila* (Zhong et al., 2019) *Bacteroides* (Zhang et al., 2013; Zhao et al., 2019) *Bacteroides uniformis* (Li et al., 2020) *Bacteroides stercoris* (Li et al., 2020) *Blautia* (Diener et al., 2021; Gaike et al., 2020) *Clostridium coccoides* (Chen et al., 2019) *Clostridium bartletti* (Zhong et al., 2019) *Clostridium hathewayi* (Zhong et al., 2019) *Clostridium leptum* (Chen et al., 2019) *Clostridium sp* (Wu et al., 2020) *Dialister invisus* (Zhong et al., 2019) *Dialister succinatiphilus* (Li et al., 2020) *Haemophilus* (Zhang et al., 2013) *Haemophilus parainfluenza* (Zhang et al., 2013; Zhong et al., 2019) *Faecalibacterium prausnitzii* (Bhute et al., 2017; Zhang et al., 2013) *Faecalibacterium sp* (Wu et al., 2020) *Roseburia* (Zhang et al., 2013) *Roseburia hominis* (Zhong et al., 2019) *Prevotella* (Zhao et al., 2019) *Prevotella copri* (Bhute et al., 2017)	*Clostridium* (Allin et al., 2018; Karlsson et al., 2013) *Clostridium botulinum* (Karlsson et al., 2013) *Clostridium beijerinckii* (Karlsson et al., 2013) *Clostridium hathewayi* (Zhong et al., 2019) *Clostridium sp* (Karlsson et al., 2013; Wu et al., 2020) *Clostridium thermocellum* (Karlsson et al., 2013) *Faecalibacterium prausnitzii* (Allin et al., 2018; Ghaemi et al., 2020; Karlsson et al., 2013; Zhong et al., 2019) *Faecalibacterium sp*. (Wu et al., 2020)	

All findings are significant (p < 0.05).

**Figure 2:**
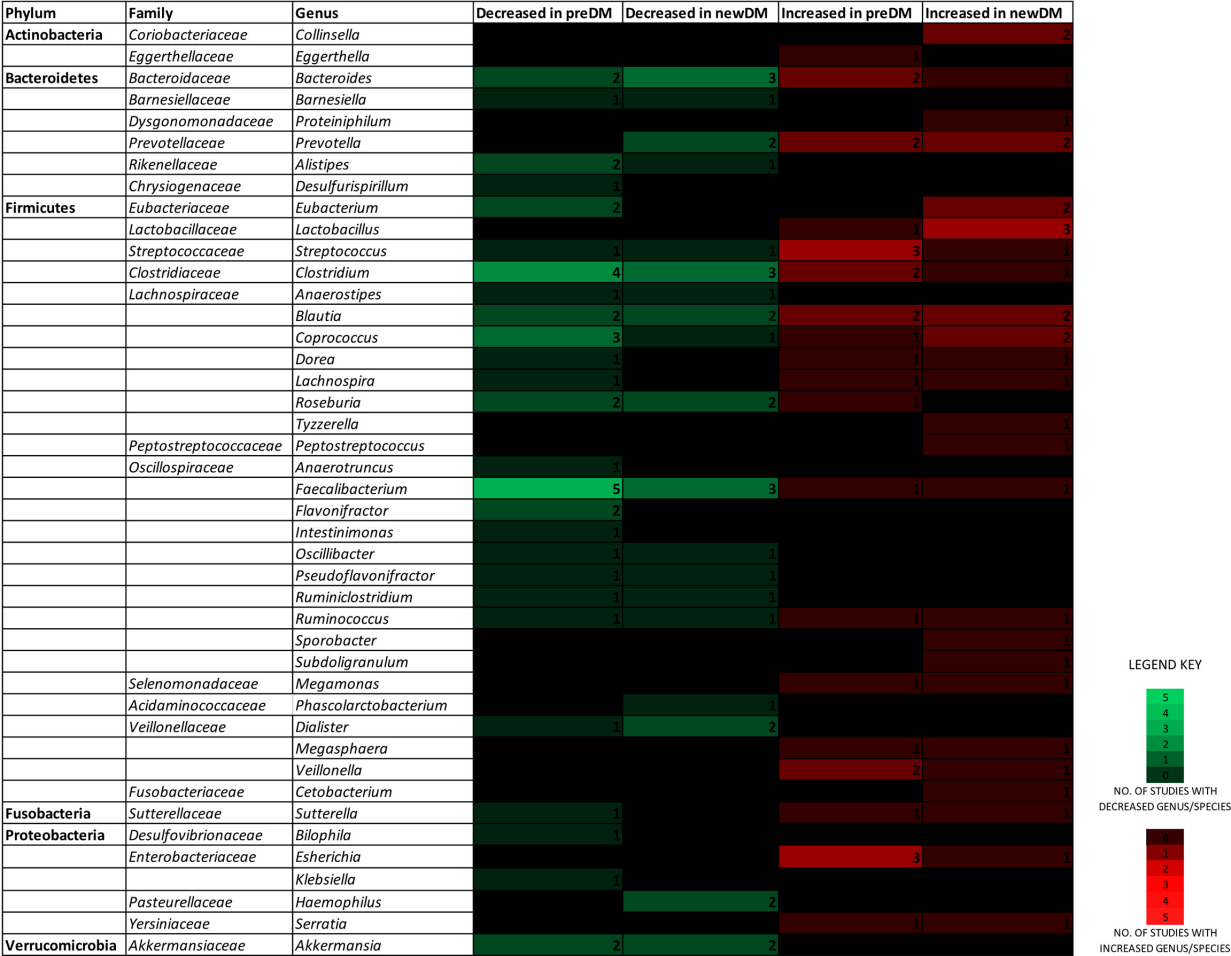
A heatmap-like view depicting the genera/species of the six predominant gut microbial phyla, found to increase or decrease in the preDM and newDM groups, by number of reporting studies.

**Figure 2** depicts findings from all 18 studies on the significantly differing genera/species belonging to the six predominant gut bacterial phyla in a heatmap-like format. When focusing exclusively on changes reported by two or more studies, the composition of a particular genera/species demonstrated distinct changes in disease groups (**Table 4** and **Table S2**). The number of *Streptococcus* (Karlsson et al., 2013; Allin et al., 2018; Zhong et al., 2019), *Escherichia* (Diener et al., 2021; Zhong et al., 2019; Ghaemi et al., 2020) and *Veillonella* (Diener et al., 2021; Nuli et al., 2019) in preDM were increased. Similarly, *Lactobacillus* (Bhute et al., 2017; Chen et al., 2019; Gaike et al., 2020) and *Collinsella* (Zhang et al., 2013; Zhong et al., 2019), were increased in newDM. On the other hand, *Faecalibacterium prausnitzii* (Karlsson et al., 2013; Allin et al., 2018; Zhong et al., 2019; Ghaemi et al., 2020; Wu et al., 2020), *Akkermansia* (Zhang et al., 2013; Allin et al., 2018), *Alistipes* (Karlsson et al., 2013; Wu et al., 2020), *Flavonifractor* (Nuli et al., 2019; Wu et al., 2020) and *Roseburia* (Karlsson et al., 2013; Zhong et al., 2019) were decreased in preDM while *Akkermansia* (Zhong et al., 2019; Gaike et al., 2020), *Dialister* (Zhong et al., 2019; Li et al., 2020), *Haemophilus* (Zhang et al., 2013; Zhong et al., 2019), *Roseburia* (Zhang et al., 2013; Zhong et al., 2019) and *Faecalibacterium* (Zhang et al., 2013; Bhute et al., 2017; Wu et al., 2020) were decreased in newDM. *Bacteroides* (Zhang et al., 2013; Karlsson et al., 2013; Allin et al., 2018; Zhao et al., 2019; Ghaemi et al., 2020; Li et al., 2020) and *Prevotella* (Zhang et al., 2013; Egshatyan et al., 2016; Bhute et al., 2017; Zhao et al., 2019) of the phylum *Bacteroidetes*; *Blautia* (Diener et al., 2021; Egshatyan et al., 2016; Allin et al., 2018; Zhao et al., 2019; Gaike et al., 2020), *Eubacterium* (Zhang et al., 2013; Karlsson et al., 2013; Zhao et al., 2019; Ericson et al., 2020), *Clostridium* (Karlsson et al., 2013; Allin et al., 2018; Chen et al., 2019; Zhong et al., 2019; Wu et al., 2020) and *Coprococcus* (Allin et al., 2018; Zhao et al., 2019; Zhong et al., 2019; Wu et al., 2020) of the phylum *Firmicutes* all exhibited changes in both directions.

### Correlation of Gut Microbiota Composition With Other Parameters

Fifteen studies assessed clinical indices and dietary habits in their study groups and correlated them with individual gut microbial taxa abundance (Diener et al., 2021; Zhang et al., 2013; Karlsson et al., 2013; Lambeth et al., 2015; Egshatyan et al., 2016; Bhute et al., 2017; Allin et al., 2018; Chen et al., 2019; Nuli et al., 2019; Zhao et al., 2019; Gaike et al., 2020; Li et al., 2020; Chávez-Carbajal et al., 2020; Ericson et al., 2020; Wang et al., 2021). These are depicted in **Tables S3, S4** respectively (**Supplementary Material**).

Nine studies reported that preDM (Zhang et al., 2013; Egshatyan et al., 2016; Allin et al., 2018; Wu et al., 2020; Ericson et al., 2020) and newDM (Zhang et al., 2013; Chen et al., 2019; Zhao et al., 2019; Wu et al., 2020) had significantly increased BMI. Five studies found a positive correlation between the increase of BMI with genera *Blautia*, *Eubacterium*, *Roseburia* (Ericson et al., 2020), *Streptococcus*, *Veillonella*, *Prevotella* (Zhao et al., 2019) or negative correlation with genera *Prevotella* (Nuli et al., 2019) and *Clostridium* (Allin et al., 2018), and specifically *Clostridium coccoides* (Chen et al., 2019). Meanwhile, the increase in FPG was inversely correlated to *Bacteroides uniformis* (Li et al., 2020) and *Prevotella copri* (Bhute et al., 2017), but positively correlated to the genus *Escherichia* (Gaike et al., 2020) and *Coprococcus comes* (Allin et al., 2018). Additionally, it was noted that FPG and/or HOMA-IR were positively associated with the genus *Lactobacillus* (Diener et al., 2021; Karlsson et al., 2013; Chen et al., 2019), *Blautia wexlerae* (Allin et al., 2018)*, Clostridium* (Allin et al., 2018) and specifically *Clostridium leptum* and *Clostridium coccoides* (Chen et al., 2019). Similarly, both FPG and HbA1c were observed to either correlate positively (Zhao et al., 2019) or negatively (Gaike et al., 2020) with genus *Akkermansia*, negatively with genus *Clostridium* (Karlsson et al., 2013) and positively with genus *Lactobacillus* (Chen et al., 2019; Gaike et al., 2020) and specifically *Lactobacillus gasseri* (Karlsson et al., 2013). Besides that, it was discovered that inflammatory marker C-reactive protein was positively associated with the genus *Veillonella* (Diener et al., 2021) and negatively associated with the genus *Clostridium* (Allin et al., 2018). Likewise, interleukin-6 (IL-6) inversely correlated with the genus *Blautia* (Diener et al., 2021) whereas adiponectin was positively correlated with genus *Clostridium* (Lambeth et al., 2015).

Regarding dietary intake, five of the 15 studies assessed dietary intake using various measures (Egshatyan et al., 2016; Chen et al., 2019; Nuli et al., 2019; Chávez-Carbajal et al., 2020; Ericson et al., 2020). These included a 24-hour dietary recall (Chávez-Carbajal et al., 2020), a 3-day food record of at least 1 weekend or 1 weekday (Chen et al., 2019), a 4-day web-based food record, developed by the Swedish National Food Institute (Ericson et al., 2020), a quantitative evaluation of food intake based on a computer program ‘Analysis of Human Nutrition’ (Egshatyan et al., 2016) and a 12-month dietary recall that recorded frequency of 84 food items consumed daily, weekly, monthly, annually or never (Nuli et al., 2019).

Chen et al. (2019) reported significantly lower daily fibre intake and significantly higher fat intake in newDM when compared to the healthy controls. In newDM, they observed a positive correlation between cholesterol intake and *Clostridium coccoides* and *Clostridium leptum* while both carbohydrate and cholesterol intake were positively associated with *Bacteroides fragilis.* Fibre intake was also found to be positively associated with genus *Bifidobacterium.* On the other hand, the study noted negative correlation between carbohydrate and fat intake with family *Enterobacteriaceae* as well as between fat intake and genus *Enterococcus* (Chen et al., 2019).

Meanwhile, Nuli et al. (2019) found that daily intake of cereals, meat, salt and oil were excessive while vegetables, fish, shrimp and dairy products were insufficient in newDM when compared to nonDM. They reported the following positive correlations: energy and protein intake with genus *Prevotella*, carbohydrate intake with genus *Dialister*, cholesterol intake with genus *Megasphaera* and fibre intake with phylum *Spirochaetae* as well as negative correlation between fat intake and phylum *Actinobacteria* in both preDM and newDM (Zhang et al., 2013).

Chávez-Carbajal et al. (2020) noted increased daily energy and macronutrients intake among preDM when compared to nonDM. On the other hand, Egshatyan et al. (2016) noted higher levels of daily energy and carbohydrate consumption in newDM when compared to preDM. They also found a negative correlation between energy and cholesterol intake with *Bifidobacterium*, starch intake with *Blautia* and sugar intake with *Catenibacterium*. Meanwhile, a positive correlation was noted between starch intake with *Bifidobacterium* and carbohydrate intake with *Prevotella*. In this study, the study groups were stratified by dietary intake before analysing the association of gut microbiota with glucose intolerance. Among participants with glucose intolerance, the abundance of *Blautia* was increased even in those consuming lower carbohydrate or fat while high abundance of *Serratia* was increased among participants who consumed equal amounts of energy and carbohydrate. Regardless of energy intake per day, the glucose intolerance group had a significantly decreased abundance of the phylum *Verrucomicrobia* (Egshatyan et al., 2016).

Ericson et al. (2020) observed that a diet consisted of what they defined as a ‘health-conscious food pattern’ was associated with a lower prevalence of prediabetes. This association, according to their findings, is linked to BMI and gut microbiota, especially a higher abundance of *Roseburia*. They also found that in preDM, fibre intake had a positive association with genera *Roseburia* and *Lachnospira* and a negative association with *Eubacterium* (Karlsson et al., 2013).

## Discussion

This systematic review aimed to provide an overview of changes reported in the gut microbiota of subjects with preDM and newDM, unperturbed by pharmacotherapy, when compared to individuals with normal glucose tolerance. The comprehensive search of the databases and references list resulted in inclusion of a majority of high-quality studies that contributed to the review’s strength. We discuss herein the consistent differences in the composition of gut microbiota and their correlation with various parameters.

Bacteria in the gut predominantly belong to six phyla i.e., *Firmicutes*, *Bacteroidetes*, *Actinobacteria*, *Proteobacteria*, *Fusobacteria*, and *Verrucomicrobia*, with the two phyla *Firmicutes* and *Bacteroidetes* accounting for 90% of the total gut microbial composition (Rinninella et al., 2019). The changes in the abundance of specific *Firmicutes and Bacteroidetes* species and the overall increase or decrease in *F/B* ratio are often associated with several diseases. Studies in obesity and known T2DM have found that the F/B ratio increases (Silva et al., 2020), decreases (Levy et al., 2016; d’Hennezel et al., 2017; Parada Venegas et al., 2019) or even remains unchanged (Stojanov et al., 2020; Anhê et al., 2021). In the present review, a significant increase in *Firmicutes* along with a significant decrease in *Bacteroidetes* among newDM were observed (Bhute et al., 2017; Nuli et al., 2019; Zhao et al., 2019; Gaike et al., 2020). However only three (Zhao et al., 2019; Gaike et al., 2020; Li et al., 2020) out of the 18 studies reported on the F/B ratio, even so with contradictory findings. In past studies, an increase in *Firmicutes* or a higher *F/B* ratio has been linked with development of obesity, as *Firmicutes* are more efficient than *Bacteroidetes* in harvesting energy from food, thus contributing to the extra calories (Magne et al., 2020). While obesity is a major risk factor for T2DM, the disease is also characterized by a state of low-grade inflammation that precedes the onset of glucose intolerance. The pro-inflammatory cytokines are said to impair insulin signalling, increase permeability and inflammation in the intestinal epithelium, eventually leading to development of insulin resistance (Tamanai-Shacoori et al., 2017). While the notion remains that this continuous low-grade inflammatory state is caused by lipopolysachharide (LPS) produced by the Gram-negative gut microbiota (Cani et al., 2007), it is now evident that *Bacteroidetes*, the most abundant group of Gram-negative bacteria in the gut produces distinct subtypes of LPS with immunoinhibitory functions that prevents inflammation (d’Hennezel et al., 2017). Logically, a decrease in either of the two dominant *Firmicutes* or *Bacteroidetes* phyla, could possibly increase the relative abundance of other Gram-negative bacteria such as those belonging to the phylum *Proteobacteria*, hence inducing production of more pro-inflammatory LPS subtypes (Magne et al., 2020; Anhê et al., 2021). An increase in other phyla, however, may not necessarily further affect the F/B ratio (Stojanov et al., 2020). Moreover, the F/B ratio does not consider compositional changes that may be occurring in the larger variety of family, genus and species taxonomic levels of each phylum. The relevance of the *F/B* ratio to serve as a disease marker for metabolic diseases is therefore inconclusive. Another important microbiota-associated factor in health and disease is the production of short-chain fatty acids (SCFAs). SCFAs, namely acetate, propionate and butyrate are metabolic products of fibre fermentation by bacteria in the gut. They are shown to exert many beneficial effects on human metabolism and immune system (Parada Venegas et al., 2019; Silva et al., 2020). *Firmicutes* are the primary producers of butyrate while *Bacteroidetes* mainly produce acetate and propionate (Levy et al., 2016; Parada Venegas et al., 2019). Gut microbiota dysbiosis has been shown to alter SCFA production, thereby affecting the epigenetic regulation of genes modulating insulin resistance and inflammatory reactions seen in T2DM (Remely et al., 2014). In relation to this, although an increase in phyla *Firmicutes* was noted, several members of this phyla were constantly found to be decreased in the disease groups. They were *Faecalibacterium prausnitzii*, *Roseburia*, *Dialister* and *Flavonifractor*. The role of these organisms as biomarkers of health is well established. Their beneficial effects are mainly attributed to their ability to produce SCFAs, especially butyrate, that play a major role in maintaining intestinal barrier integrity, energy homeostasis, attenuating inflammation and modulating glycaemic response (Tamanai-Shacoori et al., 2017; Martín et al., 2018; Mukherjee et al., 2020). Previous studies also found *F. prausnitzii* (Graessler et al., 2013; Remely et al., 2014; Candela et al., 2016), *Dialister* (Almugadam et al., 2020) and *Roseburia* (Forslund et al., 2015) to be reduced among known T2DM patients on medication. Taken together, these findings indicate that these bacteria are depleted in the gut microbiome prior to the onset of diabetes and may remain so even after treatment is initiated.

Other bacteria noted to be decreased in the disease groups in this review were *Alistipes* of phylum *Bacteroidetes*, *Akkermansia muciniphila*, of phylum *Verrucomicrobia* and *Haemophilus*, of phylum *Proteobacteria*. *Alistipes*, however was found to be increased in abundance among known T2DM patients (Wu et al., 2010; Qin et al., 2012). Indeed this bacterium is known to have protective effects against certain diseases including cardiovascular disease, while also being pathogenic in others due to its inflammatory potential (Parker et al., 2020). Previous studies showed that the mucin-degrading and gut barrier protecting *A. muciniphila* improve glucose tolerance in high fat diet-induced diabetic mice upon metformin treatment (Shin et al., 2014; de la Cuesta-Zuluaga et al., 2017). On the other hand, a decrease in abundance of this bacterium increases gut permeability, a known characteristic in T2DM progression (Cani et al., 2008). The decrease in *Haemophilus* is in agreement with a study investigating the gut microbiota pattern in women with active vs sedentary lifestyle that noted an increase in this genus, along with other health-promoting bacterial species *Faecalibacterium prausnitzii*, *Roseburia hominis* and *Akkermansia muciniphila* in active women (Bressa et al., 2017). This is unexpected given that the genus *Haemophilus* is a mucosal pathogen and its abundance has been associated with varying pathogenicity in infections (Nørskov-Lauritsen, 2014), multiple sclerosis (Chen et al., 2016) and colorectal carcinoma (Liu et al., 2020).

On the other hand, the bacteria found to have increased in abundance among the disease groups included *Firmicutes*: *Streptococcus*, *Veillonella* and *Lactobacillus*, specifically *L. ruminis*, *L. gasseri* and *L.salivarius as well as Escherichi*a (a *Proteobacteria*) and *Collinsella* (an *Actinobacteria*). *Streptococcus*, *Escherichia* and *Collinsella* are all known gut inhabitants whose abundance are associated with several inflammatory diseases including T2DM (Qin et al., 2012; Candela et al., 2016). Increased abundance of *Escherichia coli* is also linked with increased microbial infections in diabetic patients (Wiwanitkit, 2011). All three genera are positively associated with animal-based diet consumption and studies have reported that these bacteria can be successfully reduced through fibre-rich and plant-based dietary interventions (Candela et al., 2016; van Soest et al., 2020). Bacteria belonging to genus *Veillonella* are Gram-negative bacteria (unlike most *Firmicutes*) (Megrian et al., 2020), however there is little evidence for their role in health and disease to date. The immunomodulatory and probiotic properties of *Lactobacillus* are well established. However, along with its association seen herein with preDM and newDM, the present review also found noteworthy positive association between the genus *Lactobacillus* and glycaemic markers including FPG, HbA1c and the associated HOMA-IR index. The abundance of *Lactobacillus* has also been linked to chronic inflammation seen in known diabetic subjects (Zeuthen et al., 2006; Larsen et al., 2010; Lê et al., 2013). Although probiotic strains of *Lactobacillus* have been found to have beneficial anti-diabetic effects in mouse models (Yun et al., 2009; Park et al., 2015; Lee et al., 2018), it is likely that the effects of endogenous *Lactobacillus* species towards health and disease is strain dependent and further studies are required to investigate their direct effect on T2DM.

Overall, the increased levels of glycaemic and pro-inflammatory markers along with low diversity of gut bacteria among the preDM and newDM generally observed in this review suggest the possibility of gut bacteria- associated inflammation-induced environment preceding the development of T2DM. Although the studies included in this review found significant association between clinical biomarkers and the abundance of specific bacterial groups, no consistent findings were observed between studies, with the exception of the correlation between glycaemic markers and *Lactobacillus* abundance. Similarly, no consistent findings between studies were noted in correlation between dietary intake and specific gut bacterial composition. However, the studies were able to conclude that a less healthy food pattern including increased carbohydrate, fat or energy intake or a reduced fibre intake correlated with prevalence of preDM or newDM and differences in dietary intake caused significant variation in specific bacterial abundance.

### Limitations

One of the limitations of the present review is that it only summarises studies investigating gut microbiota based on composition found in faecal samples. There is a need for more studies to look into microbiota of mucosal biopsies in addition to faeces, in order to differentiate mucosa-associated bacteria from the total composition present in faeces. This is because mucosa-associated bacteria may have a more pertinent role in the pathogenesis of disease. It is also important to emphasise that findings from observational studies selected herein do not conclusively establish whether changes seen in microbiota composition were a cause or effect of glucose intolerance. Hence, bacteria found to be decreased or increased in the disease groups could not be conclusively termed as being ‘protective’ or ‘pro-diabetic’, respectively.

Studies involving taxonomic analyses of microbiota have several biases. One of it is that compositional analyses of microbiota requires that the microbial proportions to be summed to a 100% total, thus reporting the relative abundance and not the absolute composition of the microorganisms present in the faecal samples of participants. Thus, if one bacterial group appears to be reduced, the others will naturally appear to be increased and vice versa. Differences in sampling fractions may also tend to introduce false positives and false negatives (Lin and Peddada, 2020).

Another challenge in analysing gut microbiome data across studies is the biases caused by the heterogenous methodology adapted. This limits the comparability of studies and leads to ambiguous results across similar studies. A meta-analysis was also not workable for the same reason. Although all studies targeted the 16S rRNA gene, factors that differed across the studies i.e. the choice of sequencing region, pipelines and databases used for bioinformatics analysis influenced the results produced. Moreover, the usage of different diversity indexes and statistical analysis affects the comparability of microbial diversity between the studies. The choice of the diversity index affects the interpretation of the microbiome data and leads to a lack of generalizable results across the studies. Besides methodological limitations, the use of alpha diversity as a biomarker in health and disease may be confounded by colonic transit time (Roager et al., 2016) and stool consistency (Falony et al., 2016).

More importantly, the taxonomic diversity exhibited by these analyses does not take into account, the functional redundancy among members of the microbiota. Taxonomically distinct bacterial species are able to perform similar metabolic functions and as such, taxonomic variation does not reflect functional variation (Louca et al., 2018). Therefore, profiling of the microbial metabolic function would be of more significance to assess impact of the microbiota on the human host in health and disease.

## Conclusion

The 18 studies included herein were found to have heterogeneity in methodology and inconsistencies in the findings on gut microbial changes observed among preDM and newDM when compared to nonDM. By focusing on changes that were similarly reported in two or more studies, it was evident that certain bacteria were found to be increased (*Lactobacillus, Streptococcus*, *Escherichi*a, *Veillonella* and *Collinsella*) and/or decreased (*Faecalibacterium prausnitzii*, *Roseburia*, *Dialister*, *Flavonifractor, Alistipes, Haemophilus* and *Akkermansia muciniphila)* in preDM and newDM. These alterations were however not consistent across all studies included, hence emphasising the uncertainty that lies in this field of study. The increased presence of *Lactobacillus* in preDM and newDM along with its positive correlation with glycaemic markers were also inconsistent observations. This renders a need for more investigation on the species/strain-specific role of this genus in T2DM disease progression and glucose regulation mechanism. Healthier food intake inversely correlated with prevalence of preDM and newDM, while differences in dietary intake caused significant variation in specific bacterial abundances. More studies should investigate the correlation of clinical biomarkers and dietary intake with gut bacterial composition in prediabetes and early T2DM to establish more consistent associations.

## Author Contributions

SP and NA conceived the review protocol. GL and SP carried the systematic searches and extracted the data. GL, NB and NA performed the quality assessment of the selected papers. MM, NB, MO, FM, FA, NM, ZI and SP critically checked the extracted data. GL wrote the first draft of the manuscript. SP reviewed and edited the entire manuscript. MM, NA and BL critically reviewed the final manuscript. All authors contributed to writing and revising the manuscript.

## Funding

This systematic review was conducted as part of a study funded by the Fundamental Research Grant Scheme from the Ministry of Education, Malaysia (FRGS/1/2018/SKK08/USIM/02/1). Geetha Letchumanan is supported by the same grant.

## Conflict of Interest

The authors declare that the research was conducted in the absence of any commercial or financial relationships that could be construed as a potential conflict of interest.

## Publisher’s Note

All claims expressed in this article are solely those of the authors and do not necessarily represent those of their affiliated organizations, or those of the publisher, the editors and the reviewers. Any product that may be evaluated in this article, or claim that may be made by its manufacturer, is not guaranteed or endorsed by the publisher.
