# Supplementary material for: Gut Microbiota Composition in Prediabetes and Newly Diagnosed Type 2 Diabetes: A Systematic Review of Observational Studies
Source: Front Cell Infect Microbiol. 2022 Aug 15;12:943427. doi: 10.3389/fcimb.2022.943427 (PMC9422273; doi:10.3389/fcimb.2022.943427)
Supplement: Supplementary file 1 [file Table_1.docx]

Supplementary Material

# Supplementary Tables

Table S1: Quality assessment

| **(a)Newcastle-Ottawa Scale for assessing the quality of case-control studies** | | | | |
| --- | --- | --- | --- | --- |
| **Studies** | **Selection** | **Comparability** | **Exposure** | **Total** |
|  | Definition and selection of cases and controls | of cases and controls | Blinding, same method, rel. abundances as outcome, complete data |  |
|  | (max=4*) | (max=2*) | (max=3*) | (max=9*) |
| Allin et al., 2018 | **** | ** | ** | 8 |
| Bhute et al., 2017 | **** | * | ** | 7 |
| Chen et al., 2019 | **** | * | ** | 7 |
| Gaike et al., 2020 | **** | * | ** | 7 |
| Ghaemi et al., 2020 | **** | * | ** | 7 |
| Lambeth et al., 2015 | **** | ** | ** | 8 |
| Li et al., 2020 | **** | ** | ** | 8 |
| Nuli et al., 2019 | **** | ** | ** | 8 |
| Wang et al., 2021 | **** | ** | *** | 9 |
| Zhang et al., 2013 | **** | * | ** | 7 |
| Zhao et al., 2019 | **** | ** | ** | 8 |
| Zhong et al., 2019 | **** | ** | ** | 8 |

| **(b): Newcastle-Ottawa Scale for assessing the quality of cross-sectional studies** | | | | |
| --- | --- | --- | --- | --- |
| **Studies** | **Selection** | **Comparability** | **Outcome** | **Total** |
|  | True and/or somewhat representatives of the outcome of interest, sample size, exposure and risk factor assessment | +/- for baseline characteristics, controlling for additional factors | Assessment of outcome and statistical test |  |
|  | (max=5*) | (max=2*) | (max=2*) | (max=9*) |
| Chávez-Carbajal et al., 2020 | *** | ** | ** | 7 |
| Diener et al., 2021 | **** | ** | ** | 8 |
| Egshatyan et al., 2016 | **** | ** | ** | 8 |
| Wu et al., 2020 | **** | ** | ** | 8 |

| **(c): Newcastle-Ottawa Scale for assessing quality of cohort studies** | | | | |
| --- | --- | --- | --- | --- |
| **Studies** | **Selection** | **Comparability** | **Exposure** | **Total** |
|  | True and/or somewhat representatives on the outcome of interest, ascertainment of exposure, the outcome at baseline | +/- for baseline characteristics, controlling for additional factors | Blinding, time to follow-up, complete follow-up, bias due to missing follow-ups |  |
|  | (max=4*) | (max=2*) | (max=3*) | (max=9*) |
| Ericson et al., 2020 | **** | ** | *** | 9 |
| Karlsson et al., 2013 | **** | ** | ** | 8 |

Table S2: The gut microbiota in preDM (top) and newDM (bottom) that were increased or decreased in at least two studies, arranged to the respective taxa levels.

| **Taxa Level** | **Increased in preDM** | | | | **Decreased in preDM** | | | | |
| --- | --- | --- | --- | --- | --- | --- | --- | --- | --- |
| **Phylum** | **Actinobacteria** | **Bacteroidetes** | **Firmicutes** | **Proteobacteria** | **Actinobacteria** | **Bacteroidetes** | **Firmicutes** | **Proteobacteria** | **Verrucomicrobia** (Egshatyan et al., 2016; Zhang et al., 2013) |
| **Order** |  |  |  |  |  |  | *Clostridiales* (Allin et al., 2018; Karlsson et al., 2013; Nuli et al., 2019) |  |  |
| **Family** |  |  |  |  | *Coriobacteriaceae* (Karlsson et al., 2013; Nuli et al., 2019) |  | *Lachnospiraceae* (Allin et al., 2018; Karlsson et al., 2013)  *Ruminococcaceae* (Allin et al., 2018; Nuli et al., 2019) |  |  |
| **Genus/**  **Species** |  | *Bacteroides fragilis* (Ghaemi et al., 2020)  *Bacteroides uniformis* (Allin et al., 2018)  *Prevotella* (Egshatyan et al., 2016; Zhang et al., 2013) | *Blautia*  (Allin et al., 2018; Egshatyan et al., 2016)  *Blautia wexlerae* (Allin et al., 2018)  *Clostridium boltae* (Wu et al., 2020)  *Clostridium clostridioforme* (Karlsson et al., 2013; Wu et al., 2020)  *Streptococcus* (Allin et al., 2018)  *Streptococcus mutans* (Karlsson et al., 2013)  *Streptococcus salivarius* (Zhong et al., 2019)  *Streptococcus thermophilus*  (Allin et al., 2018)  *Veillonella* (Diener et al., 2021; Nuli et al., 2019) | *Esherichia coli* (Ghaemi et al., 2020; Zhong et al., 2019)  *Escherichia* (Diener et al., 2021) |  | *Alistipes* (Karlsson et al., 2013)  *Alistipes obesi* (Karlsson et al., 2013)  *Alistipes sp* (Wu et al., 2020)  *Bacteroides* (Allin et al., 2018)  *Bacteroides intestinalis* (Karlsson et al., 2013) | *Blautia* (Allin et al., 2018; Diener et al., 2021)  *Coprococcus* (Allin et al., 2018)  *Coprococcus eutactus* (Wu et al., 2020)  *Coprococcus sp*. (Zhong et al., 2019)  *Clostridium* (Allin et al., 2018; Karlsson et al., 2013)    *Clostridium botulinum* (Karlsson et al., 2013)  *Clostridium beijerinckii* (Karlsson et al., 2013)  *Clostridium hathewayi* (Zhong et al., 2019)  *Clostridium sp* (Karlsson et al., 2013; Wu et al., 2020)  *Clostridium thermocellum* (Karlsson et al., 2013)  *Eubacterium* (Ericson et al., 2020)  *Eubacterium eligens*  (Karlsson et al., 2013)  *Faecalibacterium prausnitzii* (Allin et al., 2018; Ghaemi et al., 2020; Karlsson et al., 2013; Zhong et al., 2019)  *Faecalibacterium sp*. (Wu et al., 2020)  *Flavonifractor* (Nuli et al., 2019)  *Flavonifractor plautii* (Wu et al., 2020)  *Roseburia* (Karlsson et al., 2013)  *Roseburia hominis*  (Zhong et al., 2019) |  | *Akkermansia muciniphila* (Allin et al., 2018; Zhang et al., 2013) |
| **Taxa Level** | **Increased in newDM** | | | | **Decreased in newDM** | | | | |
| **Phylum** | **Actinobacteria** | **Bacteroidetes** | **Firmicutes** (Bhute et al., 2017; Gaike et al., 2020; Nuli et al., 2019; Zhao et al., 2019) | **Proteobacteria** (Gaike et al., 2020; Zhao et al., 2019) | **Actinobacteria** | **Bacteroidetes** (Bhute et al., 2017; Gaike et al., 2020; Nuli et al., 2019; Zhao et al., 2019) | **Firmicutes** | **Proteobacteria** (Bhute et al., 2017; Nuli et al., 2019) | **Verrucomicrobia** |
| **Order** |  |  |  |  |  | *Bacteroidales* (Li et al., 2020; Nuli et al., 2019) |  |  |  |
| **Family** |  |  | *Lachnospiraceae* (Nuli et al., 2019; Zhang et al., 2013) |  |  |  |  |  |  |
| **Genus/Species** | *Collinsella*  (Zhang et al., 2013)  *Collinsella intestinalis* (Zhong et al., 2019) | *Prevotella* (Egshatyan et al., 2016; Zhang et al., 2013) | *Blautia* (Egshatyan et al., 2016; Zhao et al., 2019)  *Lactobacillus* (Bhute et al., 2017; Chen et al., 2019; Gaike et al., 2020)  *Lactobacillus ruminis* (Bhute et al., 2017)  *Coprococcus 1* (Zhao et al., 2019)  *Coprococcus eutactus* (Zhong et al., 2019)  *Eubacterium* (Zhang et al., 2013)  *Eubacterium halii* (Zhao et al., 2019) |  |  | *Bacteroides* (Zhang et al., 2013; Zhao et al., 2019)  *Bacteroides uniformis* (Li et al., 2020)  *Bacteroides stercoris* (Li et al., 2020)  *Prevotella* (Zhao et al., 2019)  *Prevotella copri* (Bhute et al., 2017) | *Blautia* (Diener et al., 2021; Gaike et al., 2020)  *Clostridium coccoides* (Chen et al., 2019)  *Clostridium bartletti* (Zhong et al., 2019)  *Clostridium hathewayi* (Zhong et al., 2019)  *Clostridium leptum* (Chen et al., 2019)  *Clostridium sp* (Wu et al., 2020)  *Dialister invisus* (Zhong et al., 2019)  *Dialister succinatiphilus* (Li et al., 2020)  *Faecalibacterium prausnitzii* (Bhute et al., 2017; Zhang et al., 2013)  *Faecalibacterium s*p (Wu et al., 2020)  *Roseburia* (Zhang et al., 2013)  *Roseburia hominis* (Zhong et al., 2019) | *Haemophilus* (Zhang et al., 2013)  *Haemophilus parainfluenza* (Zhang et al., 2013; Zhong et al., 2019) | *Akkermansia* (Gaike et al., 2020)  *Akkermansia muciniphila* (Zhong et al., 2019) |

All findings are significant (p <0.050)

Table S3: Correlation between gut bacteria and clinical indices in preDM and newDM

| **ANTHROPOMETRY** | **preDM** | | **newDM** | |
| --- | --- | --- | --- | --- |
|  | **POSITIVE CORRELATION** | **NEGATIVE CORRELATION** | **POSITIVE CORRELATION** | **NEGATIVE CORRELATION** |
| **AGE** | f. *Enterococcacea* (Chávez-Carbajal et al., 2020)  g. *Faecalibacterium* (Nuli et al., 2019)  g. *Ruminococcus* (Nuli et al., 2019) | p. *Spirochaetae*  (Nuli et al., 2019) | f. *Enterobacteriaceae* (Chen et al., 2019)  g. *Faecalibacterium* (Nuli et al., 2019)  g. *Ruminococcus* (Nuli et al., 2019) | p.*Spirochaetae*  (Nuli et al., 2019)  g.*Bifidobacterium* (Chen et al., 2019) |
| **GENDER** | g.*Prevotella*  (Chávez-Carbajal et al., 2020) |  |  |  |
| **WEIGHT** |  | g.*Fusobacterium* (Chávez-Carbajal et al., 2020) |  |  |
| **BMI** | p. *Actinobacteria*  (Nuli et al., 2019)  g. *Anaerotruncus* (Ericson et al., 2020)  g. *Bifidobacterium* (Nuli et al., 2019)  g. *Blautia* (Ericson et al., 2020)  g.*Dorea* (Allin et al., 2018)  *Dorea longicatena* (Allin et al., 2018)  g. *Eubacterium* (Ericson et al., 2020)  g*. Lachnospira* (Ericson et al., 2020)  g. *Roseburia* (Ericson et al., 2020)  g.*Ruminococcus* (Allin et al., 2018) | α-diversity (Allin et al., 2018)  f.*Lachnospiracea* (Allin et al., 2018)  f.*Ruminococcaceae* (Allin et al., 2018)  g.*Clostridium* ( (Allin et al., 2018)  *Lachnobacterium bovis* ( (Allin et al., 2018)  g.*Phascolarctobacterium* (Nuli et al., 2019)  g.*Prevotella* (Nuli et al., 2019)  *Pseudoflavonifractor capillosus* (Allin et al., 2018) | p. *Actinobacteria* (Nuli et al., 2019)  f.*Prevotellaceae* (Zhao et al., 2019)  g. *Bifidobacterium* (Nuli et al., 2019)  g.*Prevotella* (Zhao et al., 2019)  g.*Pseudobutyrivibrio* (Zhao et al., 2019)  g.*Streptococcus* (Zhao et al., 2019)  g. *Veilonella* (Zhao et al., 2019)  g.*Weisella* (Zhao et al., 2019) | f.*Ruminococcaceae* (Nuli et al., 2019)  *Clostridium coccoides* (Chen et al., 2019)  g*.Phascolarctobacterium* (Nuli et al., 2019)  g.*Prevotella*  (Nuli et al., 2019) |
| **WAIST CIRCUMFERENCE** | p. *Actinobacteria* (Nuli et al., 2019)  g. *Bifidobacterium* (Nuli et al., 2019)  *Blautia wexlerae* (Allin et al., 2018)  *Coprococcus comes* (Allin et al., 2018)  g. *Dorea* (Allin et al., 2018)  *Dorea longicatena* (Allin et al., 2018)  *Dorea sp.* (Allin et al., 2018)  g.*Ruminococcus* (Allin et al., 2018) | α-diversity (Allin et al., 2018)  f.*Lachnospiraceae* (Allin et al., 2018)  f.*Ruminococcaceae* (Allin et al., 2018)  *Bacteroides intestinalis* (Karlsson et al., 2013)  g.*Clostridium* (Allin et al., 2018)  *Lachnobacterium bovis* ( (Allin et al., 2018)  g.*Phascolarctobacteriu*m (Nuli et al., 2019)  g.*Prevotella* (Nuli et al., 2019))  *Pseudoflavonifractor capillosus* ( (Allin et al., 2018) | p. *Actinobacteria* (Nuli et al., 2019)  f.*Prevotellaceae* (Zhao et al., 2019)  g*. Bifidobacterium* (Nuli et al., 2019)  g.*Prevotella*  (Zhao et al., 2019)  g.*Pseudobutyrivibrio* (Zhao et al., 2019)  g.*Streptococcus*  (Zhao et al., 2019)  g. *Veilonella* (Zhao et al., 2019)  g.*Weisella* (Zhao et al., 2019) | g.*Phascolarctobacterium* (Nuli et al., 2019)  g.*Prevotell*a (Nuli et al., 2019) |
| **HIP CIRCUMFERENCE** | g*. Bifidobacterium* (Nuli et al., 2019) | g.Phascolarctobacterium (Nuli et al., 2019)  g.Prevotella ) (Nuli et al., 2019) | f.*Prevotellaceae* (Zhao et al., 2019)  g. *Bifidobacterium* ( (Nuli et al., 2019)  g.*Prevotella* (Zhao et al., 2019)  g.*Pseudobutyrivibrio* (Zhao et al., 2019)  g.S*treptococcus* (Zhao et al., 2019)  g. *Veilonella* (Zhao et al., 2019)  g.*Weisella*  (Zhao et al., 2019) | g.*Phascolarctobacterium* (Nuli et al., 2019)  g.*Prevotella* (Nuli et al., 2019) |
| **WAIST-HIP RATIO** |  |  | f.*Prevotellaceae* (Zhao et al., 2019)  g.*Prevotella* (Zhao et al., 2019)  g.*Pseudobutyrivibrio* (Zhao et al., 2019)  g.*Streptococcu*s (Zhao et al., 2019)  g. *Veilonell*a (Zhao et al., 2019)  g.*Weisella*  (Zhao et al., 2019) |  |
| **BIOCHEMICAL PARAMETERS** | | | | |
| **Fasting blood glucose** | p. *Synergistete*s (Nuli et al., 2019)  *Blautia wexlerae* (Allin et al., 2018)  *Coprococcus comes* (Allin et al., 2018)  g. *Dorea*  (Allin et al., 2018)  *Dorea sp*. (Allin et al., 2018)  g. *Lactobacillus* (Karlsson et al., 2013)  *Lactobacillus gasseri* (Karlsson et al., 2013)  g.*Ruminococcus* (Allin et al., 2018) | α-diversity (Allin et al., 2018)  f.*Lachnospiracea* (Allin et al., 2018)  f.*Ruminococcaceae* (Allin et al., 2018)  g.*Clostridium*  (Allin et al., 2018; Karlsson et al., 2013)  *Lachnobacterium bovis* (Allin et al., 2018)  *Pseudoflavonifractor capillosus* (Allin et al., 2018) | p. *Synergistetes* (Nuli et al., 2019)  g.*Acidaminococcu*s (Gaike et al., 2020)  g. *Akkermansia* (Zhao et al., 2019)  g.*Escherichia* (Gaike et al., 2020)  g.*Lactobacillus* (Chen et al., 2019; Gaike et al., 2020)  g.*Megasphaera* (Gaike et al., 2020) | c.*Deferribacteres* (Nuli et al., 2019)  f. *Lachnospiraceae* (Bhute et al., 2017)  g. *Akkermansia* (Gaike et al., 2020)  *Bacteroides uniformis* (Li et al., 2020)  *Clostridium leptum* (Chen et al., 2019)  *Clostridium coccoides* (Chen et al., 2019)  g. *Enterococcus* (Chen et al., 2019)  *Phascolarctobacterium faecium* (Li et al., 2020)  *Prevotella copri* (Bhute et al., 2017)  g. *Suterrella* (Gaike et al., 2020) |
| **Insulin/ Fasting plasma insulin** | *Blautia wexlerae* (Allin et al., 2018) | α-diversity (Allin et al., 2018)  c.*Clostridia* (Karlsson et al., 2013)  f.*Lachnospiracea* (Allin et al., 2018)  f.*Ruminococcaceae* (Allin et al., 2018)  g.*Clostridium* (Allin et al., 2018; Karlsson et al., 2013)  *Bacteroides intestinalis* (Karlsson et al., 2013)  *Lachnobacterium bovis* (Allin et al., 2018)  *Pseudoflavonifractor capillosu*s (Allin et al., 2018) |  | *Bacteroides uniformis* (Li et al., 2020)  *Phascolarctobacterium faecium* (Li et al., 2020) |
| **Plasma C-peptide** | g. *Dorea*  (Allin et al., 2018)  g. *Ruminococcus* (Allin et al., 2018)  *Clostridium clostridioforme* (Karlsson et al., 2013) | α-diversity (Allin et al., 2018)  c.*Clostridia* (Karlsson et al., 2013)  f.*Lachnospirace*a (Allin et al., 2018)  f.*Ruminococcacea*e (Allin et al., 2018)  g.*Clostridium* (Allin et al., 2018; Karlsson et al., 2013)  *Lachnobacterium bovis* (Allin et al., 2018)  *Pseudoflavonifractor capillosus* (Allin et al., 2018) |  |  |
| **HbA1c** | g. *Lactobacillus* (Wu et al., 2020)  *Lactobacillus gasseri* (Karlsson et al., 2013)  g. *Ruminococcus*  (Allin et al., 2018) | g.*Clostridium* (Karlsson et al., 2013) | g. *Akkermansia* (Zhao et al., 2019)  g.*Lactobacillus* (Chen et al., 2019; Gaike et al., 2020)  g. *Megaspahaera* (Gaike et al., 2020) | g. *Akermansia* (Gaike et al., 2020) |
| **HOMA-IR** | g*. Blautia wexlerae* (Allin et al., 2018)  g*. Ruminococus* (Allin et al., 2018) | α-diversity (Allin et al., 2018)  f.*Lachnospiracea* (Allin et al., 2018)  f.*Ruminococcacea*e (Allin et al., 2018)  g.*Clostridium* (Allin et al., 2018)  *Lachnobacterium bovis* (Allin et al., 2018)  *Pseudoflavonifractor capillosus* (Allin et al., 2018) | g.*Lactobacillus*  (Chen et al., 2019) | *Clostridium coccoides* (Chen et al., 2019)  *Clostridium leptum* (Chen et al., 2019) |
| **OTHER CLINICAL INDICES** | | | | |
| **Systolic blood pressure** | g.*Dialister* (Nuli et al., 2019) | g. *Erwinia* (Chávez-Carbajal et al., 2020)  g.*Phascolarctobacterium*  (Nuli et al., 2019) | f.*Prevotellaceae* (Zhao et al., 2019)  g.*Dialister* (Nuli et al., 2019)  g.*Prevotella* (Zhao et al., 2019)  g.*Pseudobutyrivibrio* (Zhao et al., 2019)  g.*Streptococcus* (Zhao et al., 2019)  g. *Veilonella* (Zhao et al., 2019)  g.*Weisella*  (Zhao et al., 2019) | f*. Lachnospiraceae* (Zhao et al., 2019)  g.*Blautia* (Zhao et al., 2019)  g.*Marvinbryantia*  (Zhao et al., 2019)  g.*Phascolarctobacterium* (Nuli et al., 2019) |
| **Diastolic blood pressure** | g.*Dialister*  (Nuli et al., 2019) |  | f.*Prevotellaceae* (Zhao et al., 2019)  g.*Dialister*  (Nuli et al., 2019)  g.*Prevotella* (Zhao et al., 2019)  g.*Pseudobutyrivibrio* (Zhao et al., 2019)  g.*Streptococcus* (Zhao et al., 2019)  g. *Veilonella* (Zhao et al., 2019)  g.*Weisella*  (Zhao et al., 2019) | f. *Lachnospiraceae* (Zhao et al., 2019)  g.*Blautia* (Zhao et al., 2019)  g.*Marvinbryantia*  (Zhao et al., 2019)  g.*Phascolarctobacterium* (Nuli et al., 2019) |
| **Total cholesterol, TC** | p*. Synergistetes* (Nuli et al., 2019)  *Rothia mucilaginosa* (Wang et al., 2021)  *Citrobacter freundii* (Wang et al., 2021) | *Bifidobacterium bifidum* (Wang et al., 2021)  *Bacteroides stercoris* (Wang et al., 2021)  *Ruminococcus bromii* (Wang et al., 2021) | p. *Synergistetes*  (Nuli et al., 2019)  f.*Prevotellaceae* (Zhao et al., 2019)  g.*Prevotella* (Zhao et al., 2019)  g.*Pseudobutyrivibrio*  (Zhao et al., 2019)  g.*Streptococcus* (Zhao et al., 2019)  g. *Veilonella* (Zhao et al., 2019)  g.*Weisella*  (Zhao et al., 2019) | f. *Lachnospiraceae*  (Zhao et al., 2019)  g.*Blautia* (Zhao et al., 2019)  g.*Marvinbryantia* (Zhao et al., 2019)  g.*Phascolarctobacterium* (Nuli et al., 2019) |
| **Serum HDL** | p. *Tenericutes* (Nuli et al., 2019)  g*. Clostridium* (Lambeth et al., 2015)  *Parabacteroides distasonis* (Wang et al., 2021) | *Streptococcus infantis* (Wang et al., 2021)  *Veilonella atypica* (Wang et al., 2021)  *Veillonella dispar* (Wang et al., 2021) | p. *Tenericutes*  (Nuli et al., 2019)  f.*Prevotellaceae* (Zhao et al., 2019)  *Clostridium leptum* (Chen et al., 2019)  *Clostridium coccoides* (Chen et al., 2019)  g.*Megamonas* (Nuli et al., 2019)  g.*Prevotella* (Zhao et al., 2019)  g.*Pseudobutyrivibrio*  (Zhao et al., 2019)  g.*Streptococcus* (Zhao et al., 2019)  g. *Sutterella* (Gaike et al., 2020)  g. *Veilonella* (Zhao et al., 2019)  g.*Weisella* (Zhao et al., 2019) |  |
| **Serum LDL** | g.*Dialister* (Nuli et al., 2019)  *Rothia mucilaginosa* (Wang et al., 2021) | p.*Bacteroidete*s (Nuli et al., 2019) | f.*Prevotellaceae* (Zhao et al., 2019)  *Clostridium leptum* (Chen et al., 2019)  g.*Dialister* (Nuli et al., 2019)  g.*Prevotella* (Zhao et al., 2019)  g.*Pseudobutyrivibri*o (Zhao et al., 2019)  g*.Streptococcus* (Zhao et al., 2019)  g. *Veilonella* (Zhao et al., 2019)  g.*Weisella*  (Zhao et al., 2019) | p.*Bacteroidetes* (Nuli et al., 2019)  f. *Lachnospiraceae* (Zhao et al., 2019)  g.*Blautia* (Zhao et al., 2019)  g.*Lactobacillus* (Chen et al., 2019)  g.*Marvinbryantia* (Chen et al., 2019)  *Bacteroides uniformis* (Li et al., 2020)  *Phascolarctobacterium faecium* (Li et al., 2020) |
| **VLDL** |  |  | g. *Acidaminococcus* (Gaike et al., 2020) |  |
| **TC/HDL ratio** |  |  |  | f.*Enterobacteriaceae*  (Chen et al., 2019)  *Clostridium coccoides* (Chen et al., 2019) |
| **Triglycerides** | *Clostridium clostridioforme* (Karlsson et al., 2013) | c.*Clostridia* (Karlsson et al., 2013)  g.*Clostridium* (Karlsson et al., 2013)  *Ruminococcus callidus* (Wang et al., 2021)  *Lactobacillus sanfranciscensis* (Wang et al., 2021)  *Alistipes putredinis* (Wang et al., 2021)  *Megamonas unclassified* (Wang et al., 2021)  *Eubacterium rectale* (Wang et al., 2021) |  | *Bacteroides uniformis* (Li et al., 2020)  *Clostridium coccoides* (Chen et al., 2019)  *Phascolarctobacterium faecium* (Li et al., 2020) |
| **Fasting plasma triacylglycerol** |  | α-diversity (Allin et al., 2018)  f.*Lachnospiracea*  (Allin et al., 2018)  f.*Ruminococcaceae* (Allin et al., 2018)  g.*Clostridium* (Allin et al., 2018)  *Lachnobacterium bovis* (Allin et al., 2018)  *Pseudoflavonifractor capillosus* (Allin et al., 2018) | f.*Prevotellaceae* (Zhao et al., 2019)  g.*Prevotella* (Zhao et al., 2019)  g.*Pseudobutyrivibrio* (Zhao et al., 2019)  g*.Streptococcus* (Zhao et al., 2019)  g. *Veilonella* (Zhao et al., 2019)  g.*Weisella* (Zhao et al., 2019) | f*. Lachnospiraceae* (Zhao et al., 2019)  g.*Blautia* (Zhao et al., 2019)  *Clostridium coccoides* (Chen et al., 2019)  g.*Marvinbryantia* (Zhao et al., 2019) |
| **Fasting plasma hsCRP** | *Veillonella* (Diener et al., 2021) | α-diversity (Allin et al., 2018)  f.*Lachnospiracea* (Allin et al., 2018)  f.*Ruminococcaceae* (Allin et al., 2018)  g.*Clostridium*  (Allin et al., 2018)  *Lachnobacterium bovis* (Allin et al., 2018)  *Pseudoflavonifractor capillosus* (Allin et al., 2018) | *Veillonella* (Diener et al., 2021) |  |
| **Uric acid** | *Rothia mucilaginosa* (Wang et al., 2021)  *Oribacterium sinus* (Wang et al., 2021)  *Enterobacter cloacae* (Wang et al., 2021) | *Eubacterium ventriosum* (Wang et al., 2021)  *Parabacteroides distasonis* (Wang et al., 2021) |  |  |
| **Adiponectin** | g. *Clostridium* (Lambeth et al., 2015) |  |  |  |
| **Lipid peroxides** |  |  | g. *Acidaminococcu*s (Gaike et al., 2020)  g. *Escherichia*  (Gaike et al., 2020) |  |
| **GLP-1** |  |  |  | *Clostridium coccoides* (Chen et al., 2019) |
| **IL-1β** |  |  |  | *Clostridium coccoides* (Chen et al., 2019) |
| **IL-6** |  | *Blautia* (Diener et al., 2021)  *Anaerostipes* (Diener et al., 2021) |  | *Blautia*  (Diener et al., 2021)  *Anaerostipes* (Diener et al., 2021) |
| **Total antioxidant capacity** |  |  | g. *Akkermansia*  (Gaike et al., 2020)  g. *Blautia*  (Gaike et al., 2020) |  |

All findings are significant (p <0.050)

Table S4: Correlation between gut bacteria and dietary intake in preDM and newDM

| **DIETARY INTAKE** | **preDM** | | **newDM** | |
| --- | --- | --- | --- | --- |
|  | **POSITIVE CORRELATION** | **NEGATIVE CORRELATION** | **POSITIVE CORRELATION** | **NEGATIVE CORRELATION** |
| **Energy Intake** | g. *Prevotella*  (Nuli et al., 2019) | *Bifidobacterium* (Egshatyan et al., 2016) | *Bacteroides fragilis* (Chen et al., 2019)  g. *Prevotell*a (Nuli et al., 2019) | *Bifidobacterium* (Egshatyan et al., 2016) |
| **Carbohydrate intake** | g. *Dialister* (Nuli et al., 2019)  g. *Prevotella* (Egshatyan et al., 2016) |  | *Bacteroides fragilis*  (Chen et al., 2019)  g.*Dialister* (Nuli et al., 2019) | f.*Enterobacteriaceae* (Chen et al., 2019) |
| **Starch intake** | *Bifidobacterium sp* (Egshatyan et al., 2016) | g.*Blautia*  (Egshatyan et al., 2016) | *Bifidobacterium sp* (Egshatyan et al., 2016) | g.*Blautia* (Egshatyan et al., 2016) |
| **Sugar intake** |  | g.*Catenibacterium* (Egshatyan et al., 2016) |  | g.*Catenibacterium* (Egshatyan et al., 2016) |
| **Protein intake** | g. *Prevotella* (Nuli et al., 2019) |  | g. *Prevotella* (Nuli et al., 2019) |  |
| **Fat intake** |  | p.*Actinobacteria* (Nuli et al., 2019) |  | p.*Actinobacteria* (Nuli et al., 2019)  f.*Enterobacteriaceae* (Chen et al., 2019)  g.*Enterococcus* (Chen et al., 2019) |
| **Lipid intake** |  | g. *Kaistobacter* (Chávez-Carbajal et al., 2020) |  |  |
| **Daily Cholesterol intake** | g. *Megaspahera* (Nuli et al., 2019) | *Bifidobacterium* (Egshatyan et al., 2016) | *Bacteroides fragilis* (Chen et al., 2019)  *Clostridium coccoides* (Chen et al., 2019)  *Clostridium leptum* (Chen et al., 2019)  g.*Megasphaera*  (Nuli et al., 2019) | *Bifidobacterium* (Egshatyan et al., 2016) |
| **Daily Fibre Intake** | g. *Eubacterium* (Ericson et al., 2020)  g*. Lachnospira* (Ericson et al., 2020)  g.*Roseburia* (Ericson et al., 2020) | p. *Spirochaetae*  (Nuli et al., 2019) | *Bifidobacterium* (Chen et al., 2019) | p. *Spirochaetae*  (Nuli et al., 2019) |
| **Ethanol consumption** |  | *Bifidobacterium* (Egshatyan et al., 2016) |  | *Bifidobacterium* (Egshatyan et al., 2016) |
| **Foods [Cereals, Tuber crop, cereal potato and misc. grains, beans, nuts, beans, fungus and nuts** | g. *Fusobacteria*  (Nuli et al., 2019)  p.*Proteobacteria* (Nuli et al., 2019) | p. *Spirochaetae*  (Nuli et al., 2019)  g.*Ruminococcus* (Nuli et al., 2019)  g.S*ubdoligranulum*  (Nuli et al., 2019) | p.*Proteobacteria* (Nuli et al., 2019)  g. *Fusobacteria* (Nuli et al., 2019) | p. *Spirochaetae*  (Nuli et al., 2019)  g*.Ruminococcus* (Nuli et al., 2019)  g.*Subdoligranulum* (Nuli et al., 2019) |
| **Food (Fruits)** | g. *Prevotella*  (Nuli et al., 2019) | p. *Spirochaetae*  (Nuli et al., 2019) | g. *Prevotella*  (Nuli et al., 2019) | p. *Spirochaetae* (Nuli et al., 2019) |
| **Food (Dairy products, Egg, Poultry)** |  | g. *Dialister*  (Nuli et al., 2019) |  | g. *Dialister*  (Nuli et al., 2019) |
| **Food (Meats)** | g. *Prevotella* (Nuli et al., 2019) | g.*Ruminococcus* (Nuli et al., 2019) | g. *Prevotella* (Nuli et al., 2019) | g.*Ruminococcus* (Nuli et al., 2019) |
| **Food (Water, Salt Oil)** | g. *Dialister*  (Nuli et al., 2019) | g.*Fusobacteria*  (Nuli et al., 2019) | g. *Dialister*  (Nuli et al., 2019) | *Fusobacteria* (Nuli et al., 2019) |
| **Beverage intake** | g. *Megaspahera* (Nuli et al., 2019)  g*. Prevotella* (Nuli et al., 2019) | p.*Firmicutes* (Nuli et al., 2019)  g.*Subdoligranulum* (Nuli et al., 2019) | g. *Megasphaera* (Nuli et al., 2019)  g. *Prevotella* (Nuli et al., 2019) | p.*Firmicutes* (Nuli et al., 2019)  g.Subdoligranulum (Nuli et al., 2019) |
| **Vitamins (ADEK)** | g. *Megasphaera* (Nuli et al., 2019) |  | g. *Megasphaera* (Nuli et al., 2019) |  |
| **Vitamins B (B1, B2, Nicotinic Acid/B3, B6, Folic Acid/B9) & C** | p.*Actinobacteria*  (Nuli et al., 2019)  p. *Synergistete*s (Nuli et al., 2019)  g. *Prevotella* (Nuli et al., 2019)  g.*Ruminococcus* (Nuli et al., 2019) | p*. Spirochaetae* (Nuli et al., 2019) | p.*Actinobacteria*  (Nuli et al., 2019)  p. *Synergistete*s (Nuli et al., 2019)  g. *Prevotella* (Nuli et al., 2019)  g.*Ruminococcus*  (Nuli et al., 2019) | p. *Spirochaetae* (Nuli et al., 2019) |
| **Minerals (Calcium)** |  | p. *Spirochaetae* (Nuli et al., 2019) |  | p*. Spirochaetae* (Nuli et al., 2019) |
| **Minerals (Cobalt)** | g. *Megasphaera*  (Nuli et al., 2019) |  | g. *Megasphaera*  (Nuli et al., 2019) |  |
| **Minerals (Potassium)** | g. *Megasphaera* (Nuli et al., 2019) |  | g. *Megasphaera* (Nuli et al., 2019) |  |
| **Minerals (Magnesium)** | g*. Blautia* (Nuli et al., 2019)  g*.Ruminococcus* (Nuli et al., 2019) | c.*Deferribacteres*  (Nuli et al., 2019) | g. *Blautia* (Nuli et al., 2019)  g.*Ruminococcus* (Nuli et al., 2019) | c.*Deferribacteres* (Nuli et al., 2019) |
| **Minerals (Iron)** | g. *Megasphaera*  (Nuli et al., 2019)  g. *Prevotella* (Nuli et al., 2019) |  | g. *Megasphaera* (Nuli et al., 2019)  g*. Prevotella* (Nuli et al., 2019) |  |
| **Minerals (Zinc)** | g. *Prevotella* (Nuli et al., 2019) |  | g. *Prevotella* (Nuli et al., 2019) |  |
| **Minerals (Selenium)** | p. Synergistetes (Nuli et al., 2019)  g*. Prevotella* (Nuli et al., 2019) |  | p*. Synergistetes* (Nuli et al., 2019)  g. *Prevotella* (Nuli et al., 2019) |  |
| **Minerals (Manganese)** | g. *Dorea* (Nuli et al., 2019) |  | g. *Dorea* (Nuli et al., 2019) |  |
| **Amino acid (Tryptophan)** |  |  |  | g. *Akkermansia*  (Gaike et al., 2020) |
| **Amino acid (Tyrosine)** |  |  | g. *Acidaminococcus* (Gaike et al., 2020)  g. *Escherichia*  (Gaike et al., 2020) |  |
| **Amino acid (Isoleucine)** |  |  | g. *Acidaminococcus* (Gaike et al., 2020)  g. *Lactobacillus*  (Gaike et al., 2020) |  |
| **Amino acid (Leucine)** |  |  | g. *Acidaminococcus*  (Gaike et al., 2020) | g. *Akkermansia* (Gaike et al., 2020) |
| **Amino Acid (Histidine)** |  |  | g.*Akkermansia* (Gaike et al., 2020)  g.*Blautia* (Gaike et al., 2020)  g*. Prevotella* (Gaike et al., 2020)  g.*Ruminococcus* (Gaike et al., 2020) | g. *Sutterella* (Gaike et al., 2020) |
| **Cake intake** | p.*Tenericutes* (Nuli et al., 2019) |  | p.*Tenericute*s (Nuli et al., 2019) |  |
| **Alcohol drinking** |  | p.*Firmicutes* (Nuli et al., 2019)  g.*Ruminococcus* (Nuli et al., 2019)  g.*Subdoligranulum*  (Nuli et al., 2019)  g. *Dialister* (Nuli et al., 2019) |  | p.F*irmicutes* (Nuli et al., 2019)  g.*Ruminococcus*  (Nuli et al., 2019)  g.*Subdoligranulum*  (Nuli et al., 2019)  g. *Dialister*  (Nuli et al., 2019) |
| **Physical Activity** | g.*Dorea*  (Chávez-Carbajal et al., 2020) |  |  |  |

All findings are significant (p <0.050)
